# In Vivo CRISPR Screening Identifies the Glutamate Receptor GRIA2 as Promoting Peritoneal Metastasis of Gastric Cancer via Calcium‐Dependent β‐Catenin Activation

**DOI:** 10.1002/advs.202521746

**Published:** 2026-03-10

**Authors:** Jie Sun, Xu Yang, Wenchao Jiang, Chengbo Ji, Yingying Wu, Haoyu Sun, Xinyou Liu, Masami Yamamoto, Tetsuya Tsukamoto, Sachiyo Nomura, Junjie Zhao, Yuanyuan Ruan, Haojie Li, Xuefei Wang

**Affiliations:** ^1^ Department of Gastrointestinal Surgery Zhongshan Hospital Fudan University Shanghai China; ^2^ Cancer Center Zhongshan Hospital Fudan University Shanghai China; ^3^ Gastric Cancer Center Zhongshan Hospital, Fudan University Shanghai China; ^4^ Department of General Surgery Zhongshan Hospital (Xiamen) Fudan University Xiamen Fujian China; ^5^ Department of Pathology Nippon Veterinary and Life Science University Tokyo Japan; ^6^ Department of Pathology Graduate School of Medicine Fujita Health University Toyoake Japan; ^7^ Department of Gastrointestinal Surgery Graduate School of Medicine The University of Tokyo Tokyo Japan; ^8^ Department of Biochemistry and Molecular Biology School of Basic Medical Sciences Fudan University Shanghai China

**Keywords:** CRISPR screening, gastric cancer, GRIA2, GSK‐3β, peritoneal metastasis

## Abstract

Peritoneal metastasis is the most lethal manifestation of gastric cancer, with a median survival of less than one year, highlighting the need for new therapeutic targets. Through an in vivo genome‐wide CRISPR/Cas9 screen, we identified GRIA2, an AMPA‐type glutamate receptor subunit, as a key driver of peritoneal metastasis. GRIA2 promotes gastric cancer cell migration, invasion, stemness, and adhesion to mesothelial cells in a glutamate‐dependent manner. Mechanistically, glutamate activates GRIA2, enhancing its interaction with GSK‐3β and inducing calcium influx, inhibiting GSK‐3β kinase activity and stabilizing β‐catenin, thereby activating the Wnt/β‐catenin signaling pathway. Single‐cell RNA sequencing revealed that cancer‐associated fibroblasts are the primary source of glutamate in the peritoneal microenvironment, which establishes a paracrine axis that enhances GRIA2‐driven metastasis. Pharmacological inhibition of AMPA receptors with NBQX and Selurampanel suppressed peritoneal metastasis in both cell line‐derived and patient‐derived organoid xenograft (PDOX) mouse models. In clinical analysis, GRIA2 expression in peritoneal metastases correlated with the levels of β‐catenin and phosphorylated GSK‐3β (serine 9), with high GRIA2 expression predicting poor prognosis. These findings suggest that GRIA2 is a novel therapeutic target, and AMPA receptor antagonists are promising agents for treating gastric cancer peritoneal metastasis.

## Graphical abstract

We identified GRIA2 as a critical driver of gastric cancer peritoneal metastasis through in vivo CRISPR screening. Mechanistically, GRIA2‐mediated calcium influx inhibits GSK3β and activates Wnt/β‐catenin signaling, driven by glutamate from cancer‐associated fibroblasts. Pharmacological blockade of AMPA receptors suppressed metastasis in preclinical models, highlighting GRIA2 as a novel therapeutic target.

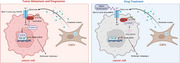

## 1 Introduction

Gastric cancer (GC) ranks as the fourth most lethal malignancy on a global scale [1]. The peritoneum represents the most common pattern of metastatic dissemination in gastric cancer and the leading site of post‐operative recurrence. Approximately 10% to 21% of patients already harbor peritoneal metastasis (PM) at initial diagnosis in population‐based studies [2, 3]. Tragically, the median survival for this condition hovers at a mere 3 to 6 months [4]. Although modern multimodal strategies—such as cytoreductive surgery combined with hyperthermic intraperitoneal chemotherapy (HIPEC)—have yielded survival benefits in selected studies [5], large randomized trials have yet to confirm meaningful improvements in overall survival [6]. Thus, current treatment paradigms appear insufficient. Enhanced knowledge of the molecular mechanisms behind peritoneal spread in gastric cancer and the discovery of implementable therapeutic targets remain essential. Despite substantial investigative effort, the molecular mechanisms governing peritoneal metastasis in gastric cancer remain only partially defined [4, 7].

Aberrant Wnt/β‐catenin signaling contributes to stem‐like traits, malignant proliferation, epithelial‐mesenchymal transition (EMT)–driven invasion, and therapy resistance [8, 9]. In the canonical Wnt pathway, the AXIN–APC–GSK‐3β destruction complex tightly controls β‐catenin stability and TCF/LEF transcriptional output [10, 11]. Within this complex, glycogen synthase kinase‐3β (GSK‐3β) serves as a principal brake: it constitutively phosphorylates β‐catenin, earmarking it for proteasomal degradation [12]. Inhibiting GSK‐3β kinase activity stabilizes β‐catenin and unleashes TCF/LEF‐dependent transcriptional programs that promote proliferative and invasive phenotypes [13, 14]. While abundant data implicate canonical Wnt/β‐catenin signaling and GSK‐3β in tumor initiation, metastasis, and therapeutic resistance, the mechanisms by which peritoneal microenvironmental cues directly inhibit GSK‐3β during GC peritoneal dissemination remain unclear.

GRIA2, also known as the glutamate ionotropic receptor AMPA type subunit 2, encodes GluR‐2, a key subunit of tetrameric AMPA receptors that mediate rapid excitatory synaptic transmission in the central nervous system [15, 16]. Aberrant GRIA2 function has been implicated in neurological disorders, including epilepsy, autism spectrum disorder, and schizophrenia [17, 18]. Of note, GluR‐2 critically determines AMPA receptor calcium permeability: edited GluR‐2 renders the channel calcium‐impermeable, whereas unedited GluR‐2–containing receptors permit calcium influx [16]. Notably, accumulating evidence suggests the expression and functional relevance of ionotropic glutamate receptors, including AMPA receptor subunits, in diverse epithelial malignancies. Prior work has shown that GRIA2 serves as a useful immunohistochemical marker for solitary fibrous tumour (SFT) [19]. More broadly, iGluR subunit expression has been detected in multiple non‐neural malignancies, including thyroid, lung, breast, colon, and gastric cancers, as well as melanoma and hepatocellular carcinoma [20, 21]. Functionally, AMPA receptors have been shown to promote tumor cell proliferation and invasion through activation of downstream signaling pathways such as MAPK [21]. In hepatocellular carcinoma, hypoxia‐driven upregulation of GRIA2 and GRIA3 enhances tumor growth [21], whereas in pancreatic cancer, AMPA receptor activation facilitates invasion and metastatic colonization, and pharmacological blockade suppresses tumor cell settlement in vivo [21, 22]. In addition, recent studies indicate that glutamate receptors can modulate the tumor microenvironment by influencing immune cell behavior [23]. These findings collectively establish a biological precedent for investigating AMPA receptor signaling as a regulator of tumor progression in non‐neural tissues, yet its role in gastric cancer peritoneal metastasis remains unexplored. Recent studies have demonstrated that Wnt/β‐catenin signaling output is sensitive to the status of the GSK‐3β‐centered destruction complex [24]. GSK‐3β activity can be downregulated through phosphorylation at Ser9 [25], as well as by the surrounding ionic milieu [26]. On this basis, we hypothesized that AMPAR‐mediated calcium influx might modulate canonical Wnt signaling in epithelial cancers, a possibility that has not been explored.

In the present study, we employed a genome‐wide CRISPR/Cas9 screen to identify genes that facilitate GC peritoneal metastasis. This unbiased survey highlighted GRIA2 as a candidate driver of peritoneal dissemination, and we revealed a mechanism linking GRIA2‐dependent calcium signaling to GSK‐3β modulation and activation of the canonical Wnt pathway. In summary, these findings shed light on the molecular mechanisms underlying gastric cancer peritoneal metastasis and unveil promising therapeutic targets for this aggressive disease.

## 2 Results

### 2.1 Genome‐wide CRISPR Screening Identifies GRIA2 as a Critical Driver of Gastric Cancer Peritoneal Metastasis

**FIGURE 1:**
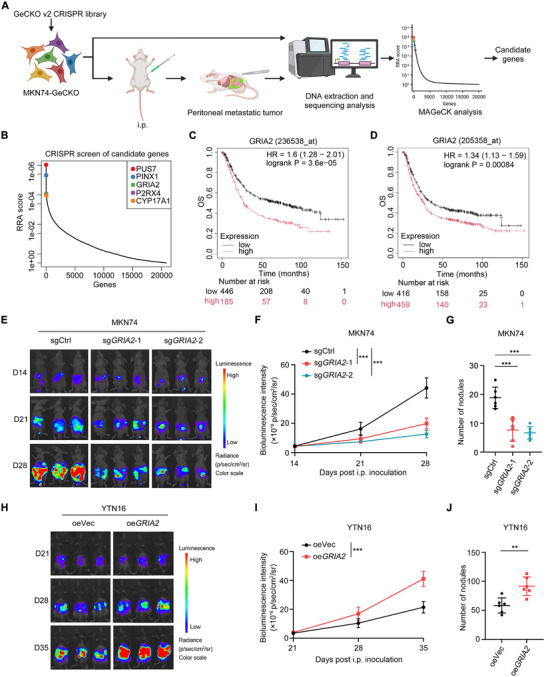
Genome‐wide CRISPR screening identifies GRIA2 as a critical driver of gastric cancer peritoneal metastasis. i.p., intraperitoneal injection (n = 36). (B) Ranked gene scores based on the robust rank aggregation (RRA) algorithm in MAGeCK analysis, with candidate genes highlighted. (C,D) Kaplan‐Meier survival analysis examining the correlation between GRIA2 expression and overall survival in gastric cancer patients from the KM plotter database. Two independent probes were analyzed: (C) probe 236538_at and (D) probe 205358_at. The optimal expression cutoff was automatically determined using the “auto select best cutoff” function in the KM plotter platform. (E) Representative bioluminescence imaging of tumor‐bearing mice at indicated time points following intraperitoneal injection of luciferase‐labeled MKN74 cells (control versus GRIA2 knockout) (n = 6 per group). (F) Quantification of bioluminescence signal intensity from mice described in (E). (G) Enumeration of peritoneal metastatic nodules in mice from the experiment shown in (E). (H) IVIS imaging assessment of tumor burden in mice following inoculation with luciferase‐ labeled YTN16 cells (control versus GRIA2 overexpression) at indicated time points (n = 6 per group). (I) Quantification of bioluminescence signal intensity at each time point in the experiment described in (H). (J) Comparison of peritoneal metastatic lesion numbers between control and GRIA2‐overexpressing groups. Two‐way ANOVA was applied to F, and I. G was analyzed by one‐way ANOVA. J was analyzed using an unpaired t‐test. Data are shown as the mean ± s.d. ^**^ *p* < 0.01, ^***^ *p* < 0.001.

To systematically identify drivers of gastric cancer peritoneal metastasis in an unbiased manner, we employed an in vivo genome‐wide CRISPR/Cas9 loss‐of‐function screen. This approach enables hypothesis‐free interrogation of gene function within the authentic peritoneal microenvironment, preserving critical tumor‐stromal interactions that are disrupted in conventional cell culture systems. In vivo CRISPR screening has been successfully applied to identify key regulators of tumor growth and metastasis [27], as well as novel targets that modulate response to cancer immunotherapy [28]. We mutagenized MKN74 cells using the human genome‐scale CRISPR knockout (GeCKO) v2 library to generate a mutant gastric cancer cell pool (MKN74‐GeCKO). Subsequently, MKN74‐GeCKO cells were employed to establish peritoneal metastasis models in athymic nude mice. We hypothesized that in the peritoneal metastasis model, cells harboring sgRNAs targeting genes that promote gastric cancer peritoneal metastasis would be selectively eliminated, leading to decreased frequency of the corresponding sgRNAs in the library. At the study endpoint, peritoneal metastases were collected. Following genomic DNA extraction from all samples, the sgRNA‐containing regions were PCR‐amplified and subjected to high‐throughput sequencing (Figure 1A). We then applied MAGeCK‐RRA computational analysis to score and rank differentially targeted genes. This analysis identified 201 significantly depleted genes (p < 0.01, log_2_ fold‐change ≤ −2, and ≥3 concordant sgRNAs). To identify potential therapeutic targets, we focused on the depleted gene set. Among our candidate hit genes, we selected PUS7, PINX1, GRIA2, P2RX4, and CYP17A1 (Figure 1B) for further investigation. Notably, GRIA2 exhibited a consistent association with poor prognosis, with two independent probes both showing significant hazard ratios (probe 236538_at: HR = 1.6, p = 3.6e‐05; probe 205358_at: HR = 1.34, p = 0.00084). Although P2RX4 and CYP17A1 also reached statistical significance, GRIA2 (probe 236538_at) demonstrated the highest hazard ratio among all candidate genes, suggesting GRIA2 might be particularly important in driving gastric cancer progression (Figure 1C,D; Figure S1A–D). Despite its established role in excitatory neurotransmission and documented expression in non‐neural neoplasms [19], the function of GRIA2 in gastric cancer peritoneal metastasis has not been investigated, prompting us to explore it further.

To validate the pro‐metastatic role of GRIA2 in gastric cancer, we first assessed GRIA2 expression levels across gastric cancer cell lines using real‐time PCR (Figure S1E). Based on the GRIA2 expression profile, we selected MKN74 and MKN45 cells, which exhibited relatively high endogenous GRIA2 expression, for knockout studies to assess loss‐of‐function phenotypes. For gain‐of‐function studies, we selected the transplantable murine gastric cancer cell line YTN16 [29], which displays low GRIA2 expression and is derived from C57BL/6 mice. We generated luciferase‐labeled cell lines with stable GRIA2 knockout (sgMKN74, sgMKN45) or overexpression (oeYTN16) (Figure S1F–H). Human gastric cancer cells (sgMKN74 and sgMKN45) were intraperitoneally injected into athymic nude mice to enable xenograft establishment, whereas murine gastric cancer cells (oeYTN16) were implanted into syngeneic C57BL/6J mice to evaluate tumor behavior within an immunocompetent peritoneal microenvironment. At designated time points, bioluminescence signal intensity of intraperitoneal tumors was monitored via IVIS imaging to evaluate the extent of tumor dissemination. Consistent with the high ranking of GRIA2 in our screen, GRIA2 ablation significantly reduced intraperitoneal bioluminescence signal intensity and the number of disseminated nodules in mice compared to those injected with control cell lines (Figure 1E–G; Figure S1I–K). Conversely, mice injected with GRIA2‐overexpressing tumor cells exhibited markedly increased intraperitoneal bioluminescence signal intensity and tumor burden (Figure 1H–J). Collectively, these data provide additional validation for our CRISPR knockout screen and confirm that increased GRIA2 expression promotes peritoneal metastasis of gastric cancer.

### 2.2 GRIA2 promotes Gastric Cancer Cell Migration, Invasion, Stemness, and Peritoneal Adhesion in a Glutamate‐Dependent Manner

**FIGURE 2:**
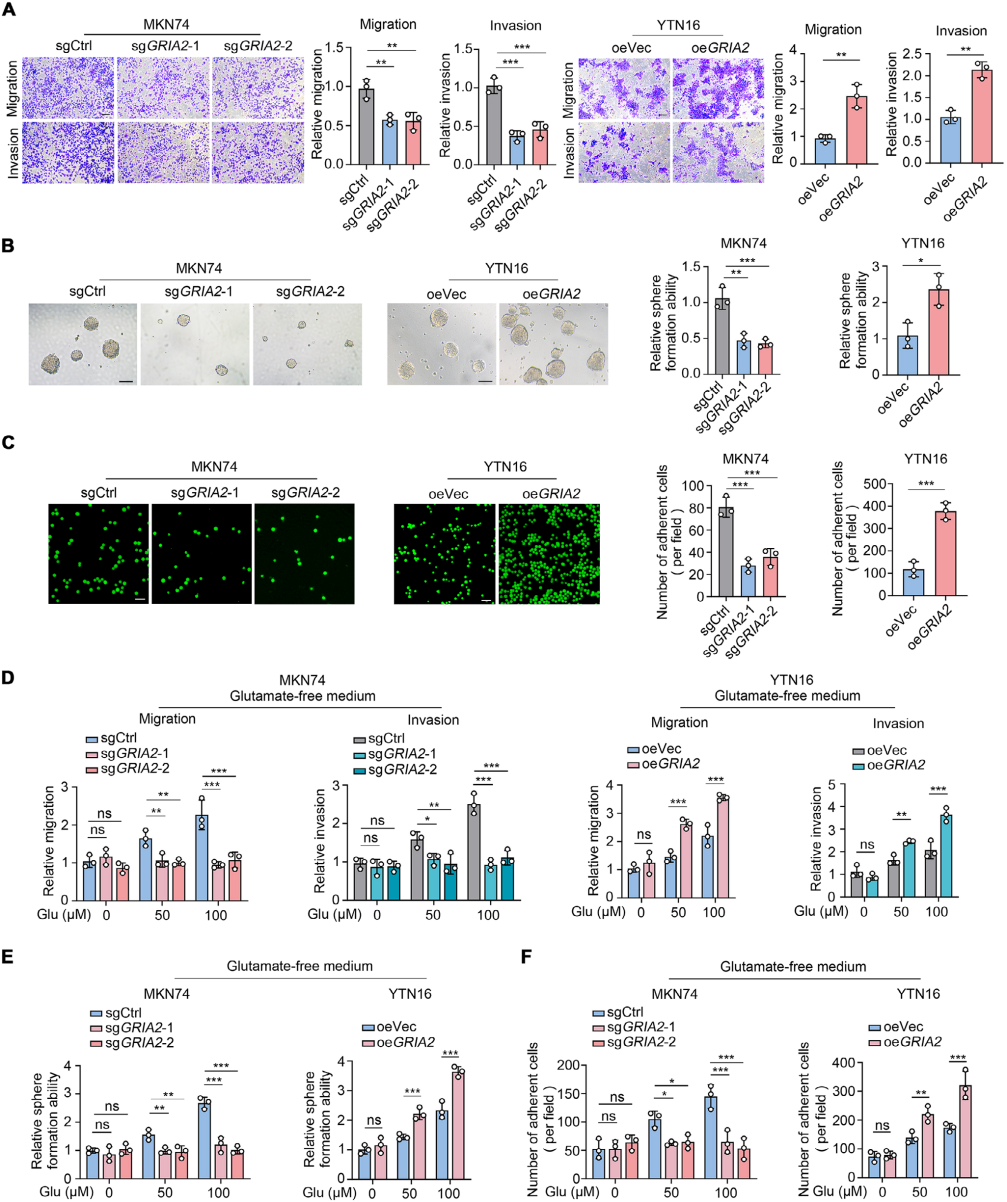
GRIA2 promotes gastric cancer cell migration, invasion, stemness, and peritoneal adhesion in a glutamate‐dependent manner. (A) Transwell assays evaluating the effects of GRIA2 knockout in MKN74 cells and GRIA2 overexpression in YTN16 cells on migration and invasion capacities, with representative images and quantitative statistical analysis (n = 3). Scale bars, 50 µm. (B) Representative images and corresponding quantification of sphere formation capacity in MKN74 cells following GRIA2 depletion and YTN16 cells after GRIA2 overexpression (n = 3). Scale bars, 150 µm. (C) Assessment of alterations in gastric cancer cell adhesion to HMrSV5 or to murine peritoneal mesothelial cells upon GRIA2 expression modulation (n = 3). Scale bars, 50 µm. (D) Dose‐dependent effects of glutamate on GRIA2‐mediated regulation of gastric cancer cell migration and invasion (n = 3). (E) Impact of varying glutamate concentrations on GRIA2‐regulated sphere formation capability (n = 3). (F) Evaluation of the influence of varying glutamate concentrations on GRIA2‐mediated adhesion of gastric cancer cells to peritoneal mesothelial cells (n = 3). Experiments in A–C were performed in standard culture medium containing basal levels of glutamine and glutamate. Experiments in D–F were performed in customized glutamine‐ and glutamate‐free medium supplemented with GlutaMAX, with exogenous L‐glutamate added at the indicated concentrations. One‐way ANOVA or unpaired t‐test was employed for A–C. Statistical significance in D–F was assessed via two‐way ANOVA. Data are shown as the mean ± s.d. ^*^ *p* < 0.05, ^**^ *p* < 0.01, ^***^ *p* < 0.001; NS, not significant.

To evaluate the biological relevance of GRIA2 expression in gastric cancer peritoneal metastasis, we first assessed the impact of GRIA2 expression on gastric cancer cell migratory and invasive phenotypes. Our data revealed that GRIA2 depletion significantly impaired the migratory and invasive capabilities of MKN74 and MKN45 cells compared to control cells (Figure 2A; Figure S2A). Conversely, ectopic GRIA2 expression substantially enhanced the migratory and invasive potential of YTN16 cells (Figure 2A). We next examined the influence of GRIA2 on the cancer stem cell (CSC)‐like phenotype of gastric cancer cells, a characteristic of self‐renewing cancer stem cells closely associated with highly metastatic tumors. We observed that loss of GRIA2 resulted in diminished sphere‐forming capacity in MKN74 and MKN45 cells, with spheroids displaying reduced size (Figure 2B; Figure S2B), whereas GRIA2 overexpression augmented sphere formation in YTN16 cells (Figure 2B). Tumor cell adhesion to peritoneal mesothelial cells serves as the initiating event in peritoneal metastasis, with augmented tumor‐mesothelial cell adhesion driving the peritoneal spread of malignant cells. Cell adhesion assays demonstrated that GRIA2 knockout reduced the adhesive capacity of MKN74 or MKN45 cells to the human peritoneal mesothelial cell line HMrSV5, whereas GRIA2 overexpression enhanced the adhesion of YTN16 cells to murine peritoneal mesothelial cells (Figure 2C; Figure S2C).

Since GRIA2 is an ionotropic glutamate receptor subunit, we next examined whether its effects on these phenotypes depended on glutamate. We further compared the impact of GRIA2 on malignant biological behaviors of gastric cancer cells under varying glutamate concentrations (0, 50, and 100 µm). Intriguingly, at 0 µm glutamate concentration, GRIA2 knockout or overexpression exerted negligible effects on migration, invasion, stem cell sphere formation, or mesothelial cell adhesion capacity of gastric cancer cells. However, with increasing glutamate concentrations in the culture medium, control gastric cancer cells progressively exhibited enhanced migration, invasion, stem cell sphere formation, and mesothelial cell adhesion capabilities (Figure 2D–F; Figure S2D–F). This phenomenon was more pronounced in GRIA2‐overexpressing cells, whereas GRIA2 depletion attenuated these effects. Taken together, our findings indicate that GRIA2 contributes significantly to the self‐renewal, migration, and adhesion of gastric cancer cells in vitro, with effects dependent on glutamate concentration.

### 2.3 GRIA2 drives Gastric Cancer Metastatic Phenotypes Through Wnt/β‐catenin Pathway Activation

**FIGURE 3:**
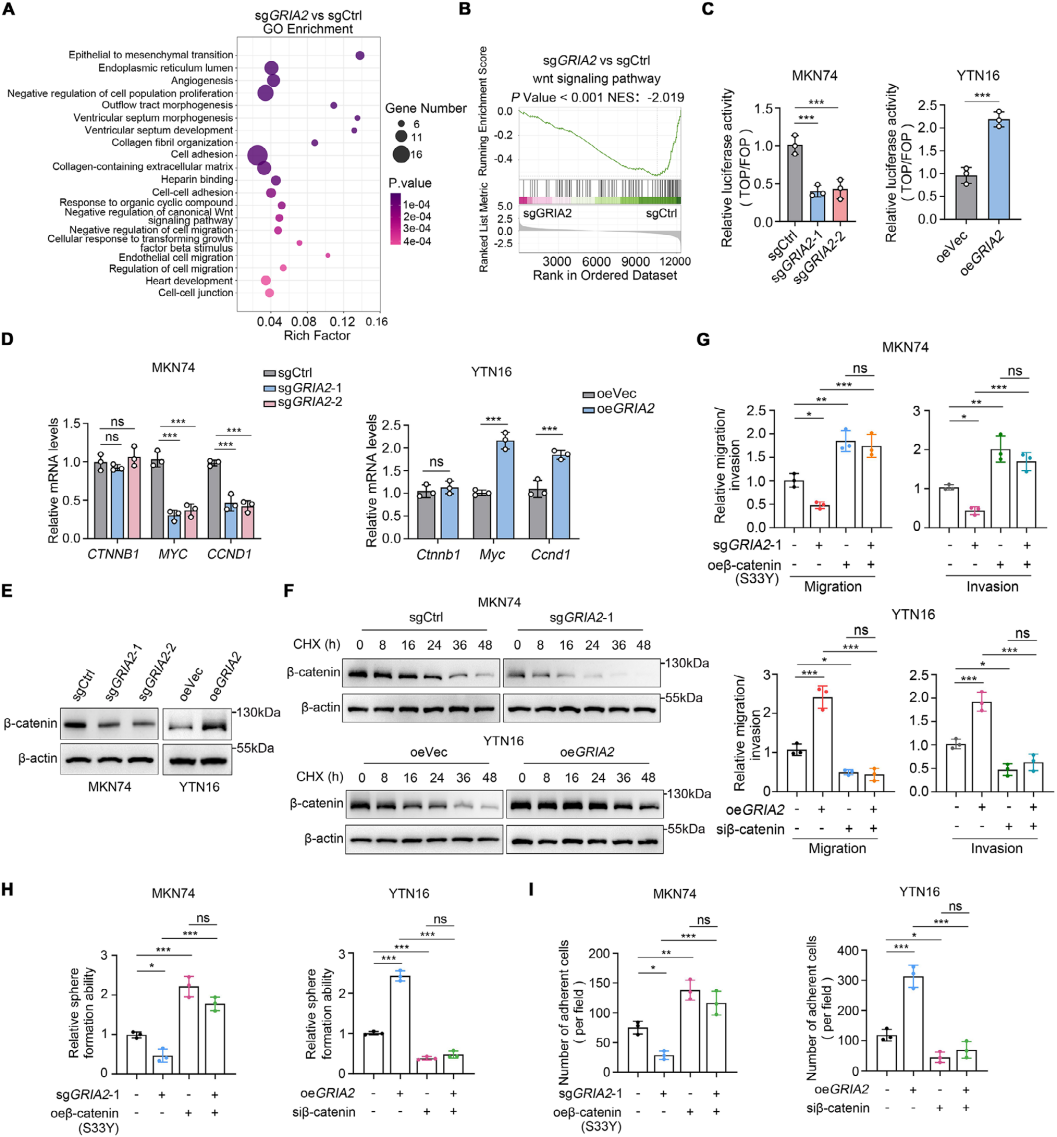
GRIA2 drives gastric cancer metastatic phenotypes through Wnt/β‐catenin pathway activation. (A) GO enrichment analysis showing the top 20 enriched terms among differentially expressed genes following GRIA2 knockout in MKN74 cells (n = 3 per group). (B) Gene Set Enrichment Analysis (GSEA) reveals significant downregulation of the Wnt signaling pathway upon GRIA2 depletion. NES, normalized enrichment score. (C) TOP/FOP luciferase reporter assay measuring transcriptional activity of the canonical Wnt pathway in MKN74 cells with GRIA2 knockout and YTN16 cells with GRIA2 overexpression (n = 3). (D) Real‐time PCR determination of *CTNNB1/Ctnnb1*, *MYC/Myc*, and *CCND1/Ccnd1* mRNA transcript levels upon GRIA2 modulation (n = 3). (E) Immunoblot analysis of β‐catenin protein expression in cells with GRIA2 knockout or overexpression. (F) Time‐course Western blot analysis of β‐catenin protein levels in MKN74 and YTN16 cells following cycloheximide (CHX, 100 µm) treatment. (G) Transwell assessment of cell migration and invasion following expression of β‐catenin mutant (S33Y) in sgCtrl and GRIA2‐depleted MKN74 cells, or transfection of β‐catenin siRNA in vector control and GRIA2‐overexpressing YTN16 cells (n = 3). (H) Analysis of the critical role of β‐catenin signaling in GRIA2‐regulated sphere formation capacity of gastric cancer cells using the experimental system described in (G) (n = 3). (I) Effects of β‐catenin (S33Y) expression in sgCtrl and GRIA2‐depleted MKN74 cells, or β‐catenin siRNA transfection in vector control and GRIA2‐overexpressing YTN16 cells, on adhesion of gastric cancer cells to peritoneal mesothelial cells (n = 3). All experiments were performed in standard culture medium containing basal levels of glutamine and glutamate. C was analyzed using one‐way ANOVA or an unpaired t‐test. D was analyzed using two‐way ANOVA. G‐I were analyzed by one‐way ANOVA. Data are shown as the mean ± s.d. ^*^ *p* < 0.05, ^**^ *p* < 0.01, ^***^ *p* < 0.001; NS, not significant.

To elucidate the molecular mechanisms underlying GRIA2‐driven gastric cancer peritoneal metastasis, we conducted RNA‐sequencing (RNA‐seq) analysis comparing GRIA2‐depleted (sgGRIA2) and control (sgCtrl) MKN74 cells. Differential expression analysis identified 336 significantly dysregulated genes, including 124 upregulated and 212 downregulated genes upon GRIA2 knockout (Figure S3A, Supplementary Table S6). GO enrichment analysis revealed that these differentially expressed genes were significantly enriched in cancer‐relevant biological processes, including epithelial‐to‐mesenchymal transition, cell adhesion, negative regulation of the canonical Wnt signaling pathway, and regulation of cell migration (Figure 3A; Table S7). Gene Set Enrichment Analysis (GSEA) further confirmed that GRIA2 depletion resulted in marked suppression of the Wnt signaling pathway (NES = ‐2.019, p < 0.001) (Figure 3B, Supplementary Table S8). The positive correlation between GRIA2 expression and Wnt signaling activity was further corroborated by data from The Cancer Genome Atlas (TCGA) gastric cancer cohort (Figure S3B). Measurement of TCF/LEF‐dependent transcriptional activity using the TOPFlash reporter system, which specifically detects canonical Wnt signal activation, demonstrated that Wnt signaling activity was substantially downregulated in GRIA2‐depleted cells and upregulated in GRIA2‐overexpressing cells (Figure 3C; Figure S3C). To examine whether GRIA2 regulates β‐catenin expression, we assessed β‐catenin mRNA and protein levels in GRIA2‐deficient and overexpressing cell lines. In these cells, GRIA2 knockout or overexpression did not affect β‐catenin mRNA levels (Figure 3D; Figure S3D), but resulted in a significant decrease or increase in β‐catenin protein expression, respectively (Figure 3E; Figure S3E), indicating that GRIA2's influence on β‐catenin occurs at the post‐transcriptional level. The activation of β‐catenin mediated by GRIA2 was further evidenced by the modulation of several β‐catenin target genes, such as *MYC/Myc* and *CCND1/Ccnd1* (Figure 3D; Figure S3D). Furthermore, time‐course treatment of gastric cancer cells with cycloheximide (CHX) revealed progressive degradation of β‐catenin under CHX treatment. GRIA2 knockout further accelerated β‐catenin degradation (Figure 3F; Figure S3F), whereas GRIA2 overexpression attenuated this degradation (Figure 3F).

We next investigated whether β‐catenin stabilization mediates the pro‐metastatic phenotypes driven by GRIA2. In MKN74 and MKN45 cells, we introduced a constitutively active β‐catenin S33Y mutant (which is resistant to GSK‐3β‐mediated phosphorylation and degradation) into both sgCtrl and sgGRIA2 groups. In YTN16 cells, β‐catenin knockdown was performed in both vector control and GRIA2‐overexpressing groups (Figure S3G). Functional studies demonstrated that β‐catenin (S33Y) overexpression rescued the impaired migration, invasion, stemness, and adhesion phenotypes of gastric cancer cells caused by GRIA2 depletion (Figure 3G–I; Figure S3H–J). Conversely, β‐catenin knockdown suppressed these malignant phenotypes in both vector control and GRIA2‐overexpressing cells, indicating that β‐catenin is essential for cancer cell migration, self‐renewal, and adhesion (Figure 3G–I). Together, these data demonstrate that GRIA2 stabilizes β‐catenin protein and activates Wnt/β‐catenin signaling, and that β‐catenin mediates GRIA2‐driven migration, invasion, self‐renewal, and peritoneal adhesion.

### 2.4 GRIA2 Physically Interacts With GSK‐3β and Inhibits Its Kinase Activity via Calcium Influx to Stabilize β‐Catenin

**FIGURE 4:**
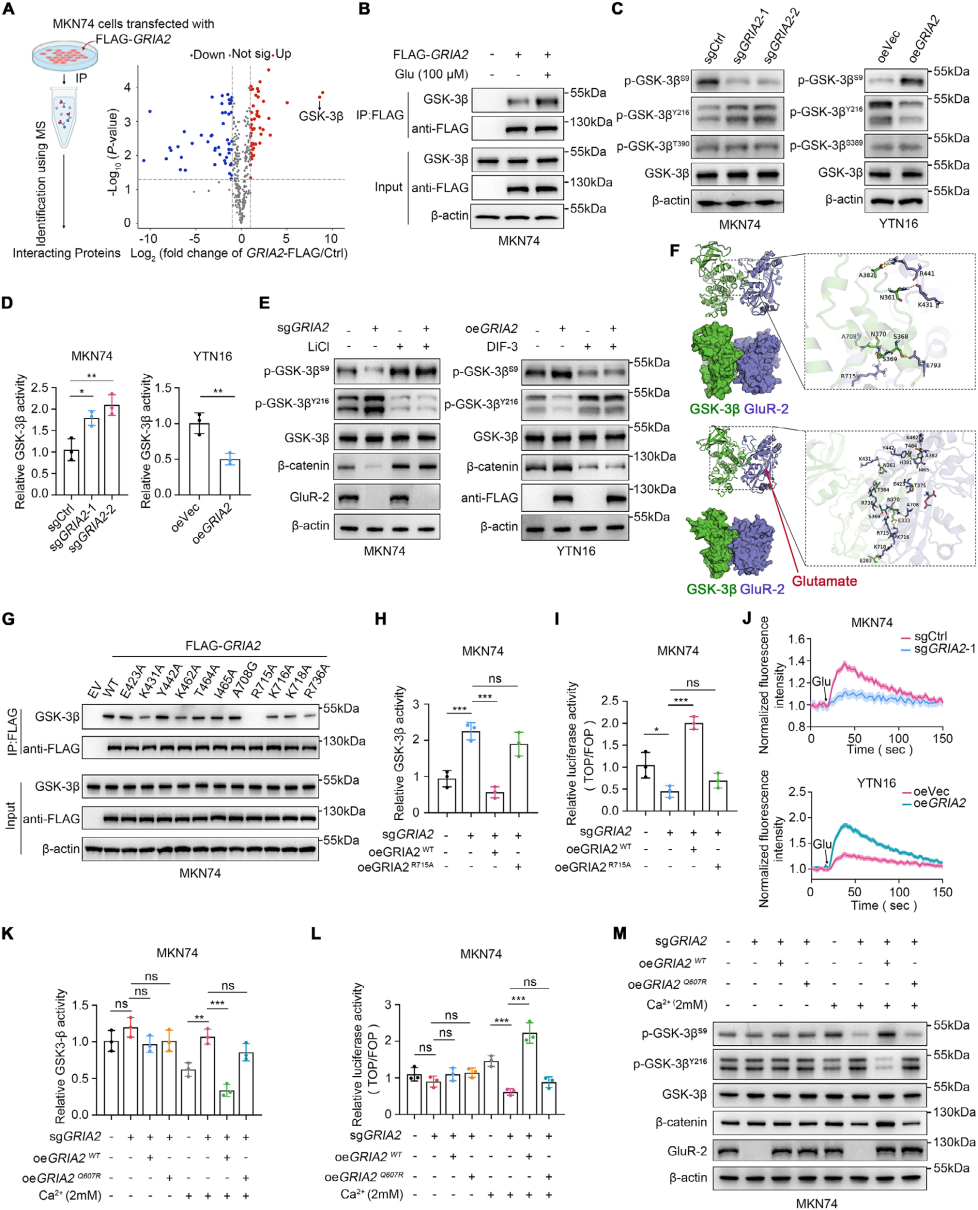
GRIA2 physically interacts with GSK‐3β and inhibits its kinase activity via calcium influx to stabilize β‐catenin. (A) Volcano plot displaying statistically significant GRIA2‐interacting proteins identified by immunoprecipitation coupled with mass spectrometry (IP‐MS) in Flag‐GRIA2‐overexpressing MKN74 cell lysates. (B) Co‐IP using Flag antibody in Flag‐GRIA2‐overexpressing MKN74 cells with or without glutamate stimulation, followed by Western blot validation of GSK‐3β binding. Input: a portion of the whole cell lysate. (C) Immunoblot assessment of the effects of GRIA2 expression modulation on p‐GSK‐3βS9, p‐GSK‐3βY216, p‐GSK‐3βS389/T390, and total GSK‐3β protein levels. (D) Kinase activity assay evaluating alterations in GSK‐3β enzymatic activity following GRIA2 knockout or overexpression (n = 3). (E) Analysis of GSK‐3β phosphorylation status, total GSK‐3β, and β‐catenin protein levels in cells treated with the GSK‐3β inhibitor LiCl (10 mm) or activator DIF‐3 (10 µm). (F) Molecular dynamics simulation illustrating the binding mode between GRIA2 (blue) and GSK‐3β (green) before and after glutamate binding. Magenta represents the glutamate molecule; yellow dashed lines indicate hydrogen bonding interactions. (G) Screening of GRIA2 point mutants constructed based on 11 key binding residues identified through molecular dynamics simulation via Co‐IP, revealing that the R715A mutation disrupts GRIA2‐GSK‐3β interaction. (H) GSK‐3β enzymatic activity measurements in GRIA2‐knockout MKN74 cells reconstituted with wild‐type GRIA2 or the R715A mutant (n = 3). (I) Measurement of TOP/FOP flash activity in MKN74 cells overexpressing wild‐type GRIA2 or R715A mutant in the GRIA2 knockout background (n = 3). (J) Quantitative analysis of calcium influx levels in MKN74 and YTN16 cells with GRIA2 knockout or overexpression using fluorescent probes. (K) GSK‐3β enzymatic activity determination in GRIA2‐knockout MKN74 cells expressing wild‐type GRIA2 or the Q607R mutant under calcium‐replete or calcium‐depleted conditions (n = 3). (L) TOP/FOP luciferase activity measurements in GRIA2‐knockout MKN74 cells overexpressing wild‐type GRIA2 or the Q607R mutant in the presence or absence of calcium (n = 3). (M) Western blot analysis of GSK‐3β phosphorylation status, total GSK‐3β, and β‐catenin protein expression. Experiments in A–E and G–I were performed in standard culture medium containing basal levels of glutamine and glutamate. For K–M, customized calcium‐free RPMI‐1640 containing basal levels of glutamine and glutamate was used, with or without CaCl_2_ (2 mM) supplementation. D was analyzed using one‐way ANOVA or an unpaired t‐test. H, I, K, and L were analyzed by one‐way ANOVA. Data are shown as the mean ± s.d. ^*^ *p* < 0.05, ^**^ *p* < 0.01, ^***^ *p* < 0.001; NS, not significant.

Next, we investigated the mechanism by which GRIA2 affects the Wnt/β‐catenin signaling pathway. We performed immunoprecipitation followed by liquid chromatography‐mass spectrometry (IP‐MS) in Flag‐GRIA2‐overexpressing MKN74 cells. The results revealed a robust association between GRIA2 and GSK‐3β (Figure 4A). Co‐immunoprecipitation (Co‐IP) experiments in both MKN74 and MKN45 cells overexpressing GRIA2 further validated this interaction between GSK‐3β and GRIA2, which was notably enhanced following glutamate treatment (Figure 4B; Figure S4A). Input (a portion of the whole cell lysate) was included to verify sample integrity and confirm antibody immunoreactivity. Having established that GRIA2 physically interacts with GSK‐3β, we next investigated whether GRIA2 modulates GSK‐3β activity. GSK‐3β activity is regulated by phosphorylation at several key residues: Ser9 phosphorylation is inhibitory, as the phosphorylated N‐terminus acts as a pseudosubstrate that blocks the substrate‐binding site [25, 30]; Tyr216 phosphorylation in the activation loop positively modulates catalytic activity [31, 32]; and Thr390 (human)/Ser389 (mouse) phosphorylation is another inhibitory site mediated by p38 MAPK [33]. Western blot analysis revealed that GRIA2 depletion decreased phosphorylation at Ser9 while increasing phosphorylation at Tyr216, with no change in total GSK‐3β levels (Figure 4C; Figure S4B). Conversely, GRIA2 overexpression produced the opposite pattern. Thus, the concurrent decrease in inhibitory p‐Ser9 and increase in activating p‐Tyr216 collectively elevate GSK‐3β activity in GRIA2‐depleted cells. Notably, Ser389/Thr390 phosphorylation remained unchanged regardless of GRIA2 status, suggesting that GRIA2 selectively regulates GSK‐3β activity through the Ser9 and Tyr216 sites rather than through Ser389/Thr390. Consistent with these findings, GSK‐3β kinase assays demonstrated that GRIA2 depletion increased GSK‐3β activity, while GRIA2 overexpression reduced GSK‐3β activity (Figure 4D; Figure S4C). In MKN74 and MKN45 cells, the GSK‐3β inhibitor LiCl increased Ser9 phosphorylation, decreased Tyr216 phosphorylation, and restored β‐catenin protein levels in both sgCtrl and GRIA2‐depleted cells (Figure 4E; Figure S4D). Conversely, in YTN16 cells, the GSK‐3β activator DIF‐3 decreased Ser9 phosphorylation, increased Tyr216 phosphorylation, and diminished β‐catenin levels in both vector control and GRIA2‐overexpressing cells (Figure 4E).

To better understand the interaction between GRIA2 and GSK‐3β and the influence of glutamate on GRIA2‐GSK‐3β binding stability, we conducted molecular dynamics simulations. Analysis revealed hydrogen bonding interactions during GSK‐3β‐GRIA2 complex formation. Upon glutamate binding, the number of hydrogen bonds between GSK‐3β and GRIA2 proteins increased, indicating enhanced binding affinity between GSK‐3β and GRIA2 in the presence of glutamate (Figure 4F). RMSD, RMSF, and radius of gyration analyses consistently demonstrated that glutamate binding enhanced the conformational stability and compactness of the GSK‐3β/GRIA2 complex (Figure S4E–G). Binding energy calculations showed that the GSK‐3β‐GRIA2‐Glu complex (−50.69 ± 3.81 kcal/mol) exhibited greater binding affinity than the GSK‐3β‐GRIA2 complex (−44.85 ± 3.04 kcal/mol) (Figure S4H). Based on these simulations, we constructed GRIA2 point mutants targeting predicted binding residues and assessed their effects on GRIA2‐GSK‐3β interaction by Co‐IP. Notably, mutation at the R715 site resulted in complete loss of GRIA2‐GSK‐3β binding ability, indicating that R715 is a key binding residue for the GRIA2‐GSK‐3β interaction (Figure 4G). GSK‐3β kinase assays showed that wild‐type GRIA2 overexpression reversed the elevated GSK‐3β activity caused by GRIA2 depletion, whereas overexpression of the GRIA2 R715A mutant did not (Figure 4H; Figure S4I). Consistently, wild‐type GRIA2 overexpression increased β‐catenin‐associated TCF/LEF transcriptional activation in the TOPFlash reporter assay, while the R715A mutant failed to do so (Figure 4I; Figure S4J).

We subsequently explored the mechanism underlying GRIA2‐mediated inhibition of GSK‐3β activity. Intriguingly, previous molecular dynamics simulations have shown that Ca^2^^+^ can displace Mg^2^^+^ at the GSK‐3β active site, thereby inhibiting its catalytic function [26]. GRIA2 undergoes A‐to‐I RNA editing at the Q/R site (Q607R), which renders the receptor calcium‐impermeable. Insufficient editing levels or increased expression of unedited glutamine (Q)‐type protein leads to enhanced calcium ion influx. Through Sanger sequencing, we observed that GRIA2 in gastric cancer cells contains unedited glutamine (Q) at position 607 (Figure S4K). Analysis of the TCGA public database showed that the average RNA editing rate of Q607R in gastric cancer patients is below 20% (Figure S4L). Therefore, we next analyzed GRIA2's regulatory effect on calcium influx in gastric cancer cells. Results showed that calcium ion influx decreased in GRIA2‐depleted gastric cancer cells and increased in GRIA2‐overexpressing cells (Figure 4J; Figure S4M). To determine whether calcium influx is required for GRIA2‐mediated regulation of GSK‐3β and Wnt/β‐catenin signaling, we constructed and expressed the GRIA2 Q607R mutant in gastric cancer cells and examined GSK‐3β activity and TCF/LEF transcriptional activity under conditions with or without calcium ions. Results demonstrated that in the presence of calcium ions, overexpression of wild‐type GRIA2 suppressed GSK‐3β activity and upregulated TCF/LEF transcriptional activity, while overexpression of the GRIA2 Q607R mutant did not induce these changes. Moreover, in the absence of calcium, neither GRIA2 knockout, wild‐type GRIA2 overexpression, nor GRIA2 Q607R mutant overexpression significantly affected GSK‐3β activity or TCF/LEF transcriptional activity (Figure 4K,L; Figure S4N,O). In calcium‐containing medium, GRIA2 knockout decreased GSK‐3β Ser9 phosphorylation and reduced β‐catenin protein levels; these changes were reversed by wild‐type GRIA2 overexpression but not by the Q607R mutant. GRIA2's regulatory effects on Ser9 phosphorylation, Tyr216 phosphorylation of GSK‐3β, and β‐catenin protein levels were eliminated in the calcium‐free system (Figure 4M; Figure S4P).

Collectively, these data establish a signaling axis in which GRIA2 binds GSK‐3β in a glutamate‐enhanced manner, and unedited GRIA2‐mediated calcium influx suppresses GSK‐3β kinase activity, leading to β‐catenin stabilization and Wnt pathway activation.

### 2.5 AMPA Receptor Antagonists Suppress Gastric Cancer Peritoneal Metastasis in Preclinical Models

**FIGURE 5:**
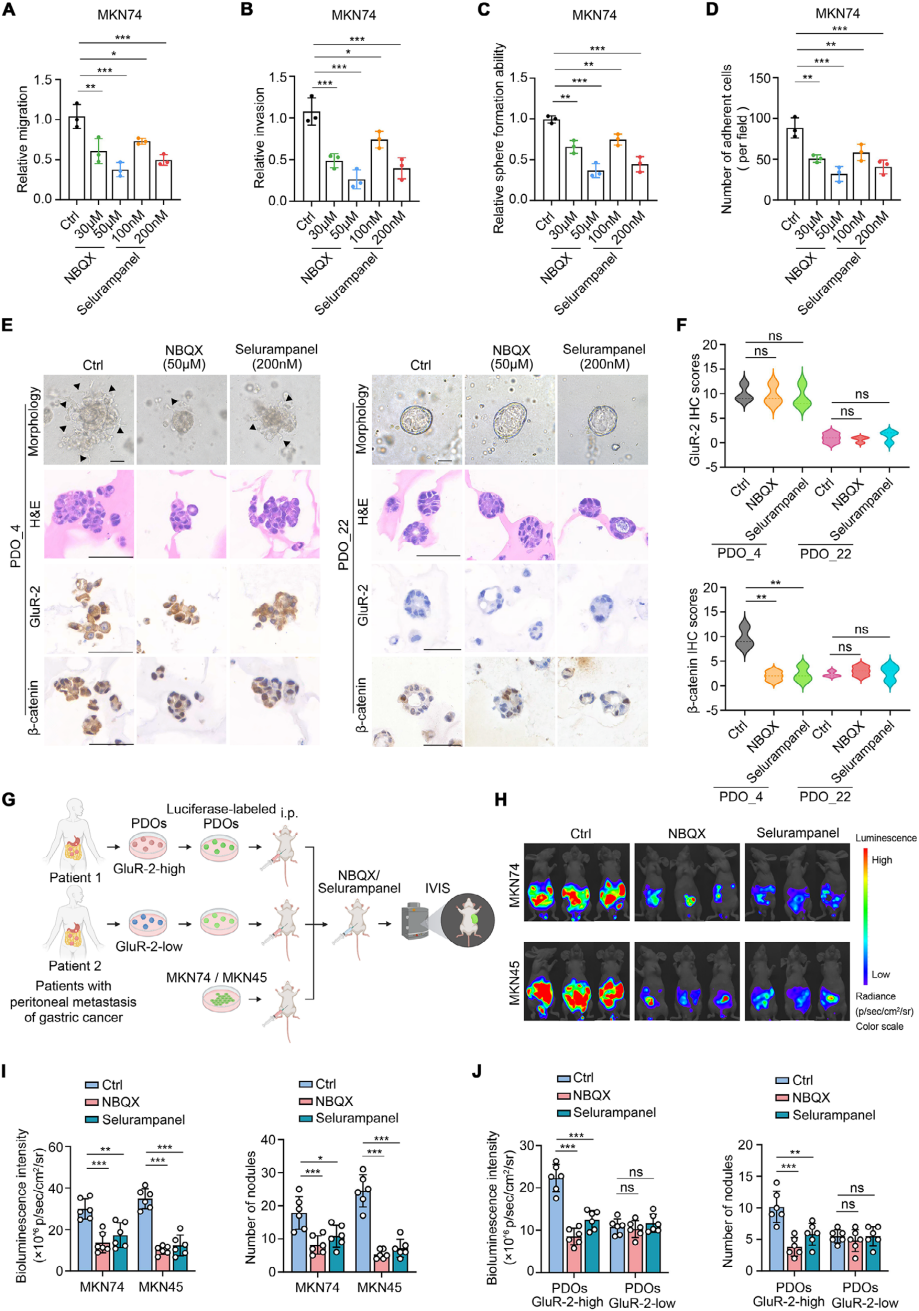
AMPA receptor antagonists suppress gastric cancer peritoneal metastasis in preclinical models. (A,B) Evaluation of MKN74 cell migration (A) and invasion (B) capacity following treatment with varying concentrations of NBQX or Selurampanel (n = 3). C,D) Effects of NBQX or Selurampanel treatment on MKN74 cell sphere formation (C) (n = 3) and adhesion to HMrSV5 cells (D) (n = 3). E) Morphological changes (bright‐field microscopy), histological characteristics (H&E staining), and GluR‐2 and β‐catenin immunohistochemical staining of patient‐derived organoids (PDOs) from gastric cancer peritoneal metastases treated with NBQX or Selurampanel. Arrows indicate invasive protrusions. Scale bars, 50 µm. (F) Quantitative analysis of GluR‐2 and β‐catenin immunohistochemical staining intensity scores in GRIA2‐high and GRIA2‐low PDOs following NBQX or Selurampanel treatment, as shown in (E). (G) Schematic of drug treatment in cell line‐derived and PDOX models. (H) Representative IVIS imaging from peritoneal metastasis models established with luciferase‐labeled MKN74 or MKN45 cells treated with NBQX or Selurampanel. (I) Quantitative analysis of bioluminescence signals and peritoneal disseminated tumor nodule counts from each group of mice in (H) (n = 6 per group). (J) IVIS signal quantification and peritoneal metastatic nodule counts from PDOX models with drug treatment (n = 6 per group). For A‐F, experiments were performed in standard culture medium containing basal levels of glutamine and glutamate. A‐D and I‐J were analyzed by one‐way ANOVA. Data are shown as the mean ± s.d. ^*^ *p* < 0.05, ^**^ *p* < 0.01, ^***^ *p* < 0.001; NS, not significant.

Given the critical role of GRIA2 in promoting peritoneal metastasis, we next investigated whether pharmacological inhibition of AMPA receptors could serve as a therapeutic strategy. We employed two selective competitive AMPA receptor antagonists, NBQX and Selurampanel. Transwell assay results demonstrated that NBQX and Selurampanel significantly impaired the migratory and invasive capabilities of MKN74 and MKN45 cells in a dose‐dependent manner (Figure 5A,B; Figure S5A,B). Similarly, compared to controls, NBQX and Selurampanel reduced sphere‐forming capacity of MKN74 and MKN45 cells (Figure 5C; Figure S5C). Additionally, we observed that NBQX and Selurampanel treatment decreased the number of MKN74 and MKN45 cells adhering to HMrSV5 cells (Figure 5D; Figure S5D).

We also explored the therapeutic potential of AMPA receptor antagonists using patient‐derived organoid (PDO) models derived from peritoneal metastatic lesions of gastric cancer patients exhibiting different GRIA2 expression levels (Table S11). Morphological examination showed that GRIA2‐high organoids developed invasive protrusions with branching structures, while GRIA2‐low organoids formed epithelial‐like spherical structures with smoother boundaries (Figure 5E). Notably, when GRIA2‐high organoids were treated with NBQX or Selurampanel, they underwent marked contraction accompanied by a substantial reduction in branching structures. By contrast, NBQX or Selurampanel had minimal impact on the volume or morphology of GRIA2‐low organoids (Figure 5E). To validate these observations at the protein level, we conducted immunohistochemistry (IHC) analysis. IHC analysis confirmed high GluR‐2 expression in GRIA2‐high organoids and low expression in GRIA2‐low organoids (Figure 5E,F). Remarkably, the expression pattern of β‐catenin closely correlated with GRIA2 levels. Treatment with NBQX or Selurampanel decreased β‐catenin expression in GRIA2‐high PDOs, whereas the same treatments had negligible effects on β‐catenin levels in GRIA2‐low PDOs (Figure 5E,F).

Finally, we evaluated the therapeutic efficacy of AMPA receptor antagonists in vivo using cell line‐derived peritoneal metastasis models and patient‐derived organoid xenograft (PDOX) models (Figure 5G). In the cell line‐derived models, IVIS imaging revealed that mice treated with NBQX or Selurampanel displayed substantially reduced bioluminescence intensity and fewer metastatic tumor nodules in the abdominal cavity compared to control groups (Figure 5H,I). In PDOX models, treatment with NBQX or Selurampanel inhibited metastatic spread of GRIA2‐high PDOs and reduced the number of metastatic nodules. In contrast, NBQX or Selurampanel did not significantly affect intraperitoneal dissemination of GRIA2‐low PDOs (Figure 5J). These data demonstrate that AMPA receptor antagonists suppress peritoneal metastasis in a GRIA2‐dependent manner, suggesting that GRIA2 may represent a potential therapeutic target.

### 2.6 Cancer‐Associated Fibroblast‐Derived Glutamate Drives GRIA2‐Mediated Peritoneal Metastasis

**FIGURE 6:**
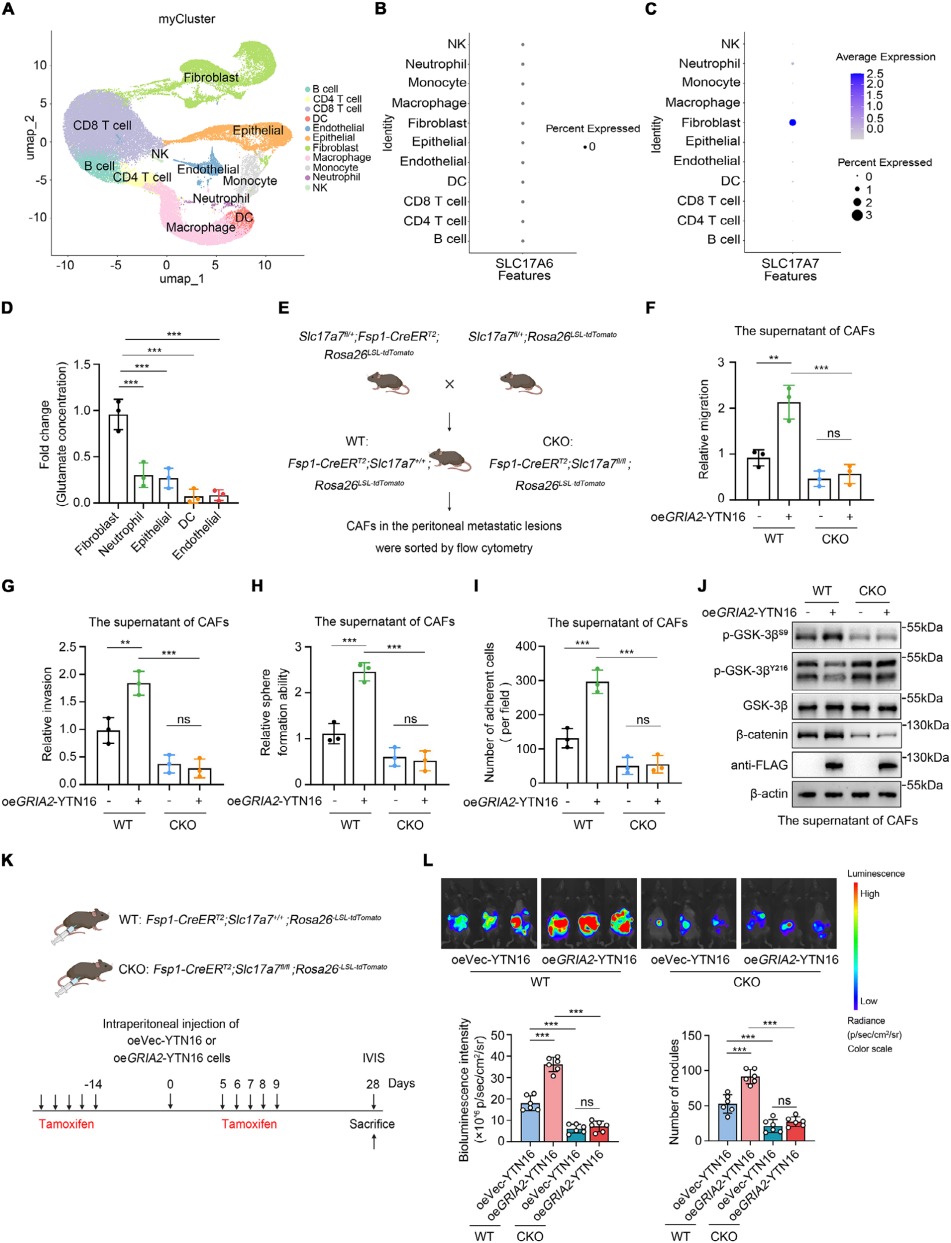
Cancer‐Associated Fibroblast‐Derived Glutamate Drives GRIA2‐Mediated Peritoneal Metastasis. (A) UMAP dimensionality reduction visualization of major cell type clusters from single‐cell transcriptome sequencing of five gastric cancer peritoneal metastasis samples. Different colors represent distinct clusters (n = 5). (B,C) Dot plots showing the expression distribution of glutamate transporters SLC17A6 (B) and SLC17A7 (C) across different cell types. (D) Extracellular glutamate levels from different cell types isolated from gastric cancer peritoneal metastases (n = 3). (E) Schematic strategy for generating fibroblast‐specific *Slc17a7* conditional knockout mice (*Slc17a7* CKO), with wild‐type littermates (*Slc17a7* WT) serving as controls. (F–I) Effects of conditioned media from WT or CKO CAFs on migration (F), invasion (G), sphere formation (H), and peritoneal adhesion (I) of oeVec‐YTN16 and oeGRIA2‐YTN16 cells (n = 3). (J) Western blot detection of GSK‐3β phosphorylation and β‐catenin protein expression following treatment with different conditioned media. (K) Experimental workflow for peritoneal metastasis models using oeVec‐YTN16 or oeGRIA2‐YTN16 cells in *Slc17a7* WT and CKO mice. (L) IVIS imaging, bioluminescence quantification, and peritoneal nodule counts from oeVec‐YTN16 or oeGRIA2‐YTN16 cells in *Slc17a7* WT and CKO mice (n = 6). For D, sorted cells were cultured in customized glutamine‐ and glutamate‐free medium supplemented with GlutaMAX. F–J were performed using conditioned media from WT or CKO CAFs. D was analyzed using one‐way ANOVA. F–I, and L were analyzed by two‐way ANOVA. Data are shown as the mean ± s.d. ^**^ *p* < 0.01, ^***^ *p* < 0.001; NS, not significant.

We also investigated the cellular sources of glutamate, the endogenous ligand for AMPA receptors, within the tumor microenvironment. We performed single‐cell RNA sequencing on five gastric cancer peritoneal metastasis specimens (Table S10) and analyzed the expression patterns of SLC17A6 (VGluT2) and SLC17A7 (VGluT1), two key glutamate transporters, across various cell types in the tumor microenvironment (Figure 6A; Figure S6A). Results showed that SLC17A6 was virtually undetectable in all cell types (Figure 6B). SLC17A7 was predominantly expressed in fibroblasts, with low or negligible expression levels in other immune and epithelial cell types (Figure 6C; Table S9). Based on scRNA‐seq data, the top five cell types expressing SLC17A7 were fibroblasts, neutrophils, epithelial cells, dendritic cells, and endothelial cells. To validate these findings, we isolated these five cell populations from human gastric cancer peritoneal metastasis tissues by fluorescence‐activated cell sorting (FACS). Real‐time PCR confirmed that fibroblasts had the highest SLC17A7 expression among the sorted cell populations (Figure S6B). Consistently, glutamate measurements revealed that fibroblasts released the greatest amounts of glutamate into the culture medium (Figure 6D). These findings suggest that fibroblasts within the tumor microenvironment (commonly termed cancer‐associated fibroblasts, CAFs) are a major source of glutamate in human gastric cancer peritoneal metastases.

We next examined whether this finding extends to mouse models. Western blot analysis demonstrated VGluT1 protein expression in CAFs isolated from mouse peritoneal metastases, with mouse brain lysate serving as a positive control (Figure S6C). Based on this finding and to better understand the functional role of CAF‐derived glutamate in gastric cancer peritoneal metastasis, we generated fibroblast‐specific *Slc17a7*‐deficient mice (CKO: *Slc17a7^fl/fl^/Fsp1‐CreER^T2^/Rosa26^LSL‐tdTomato^*) and control littermates (WT: *Slc17a7^+/+^/Fsp1‐CreER^T2^/Rosa26^LSL‐tdTomato^*) (Figure 6E). Gastric cancer peritoneal metastasis models were established using WT and CKO mice. Single‐cell suspensions were obtained from peritoneal metastatic lesions, and tdTomato‐positive cells were isolated by FACS. Real‐time PCR and Western blot analyses showed efficient depletion of *Slc17a7* in sorted tdTomato‐positive CAFs from CKO mice compared to WT controls (Figure S6D,E). Conditioned media from tdTomato‐positive WT CAFs significantly enhanced migration, invasion, sphere formation, and peritoneal adhesion of oeGRIA2‐YTN16 cells compared to oeVec‐YTN16 cells (Figure 6F–I). By contrast, in the presence of conditioned media from CKO CAFs, no significant differences were observed between oeVec‐YTN16 and oeGRIA2‐YTN16 cells in these phenotypes (Figure 6F–I). Western blot analysis showed that in the presence of WT CAFs' conditioned medium, oeGRIA2‐YTN16 cells exhibited increased GSK‐3β Ser9 phosphorylation, decreased Tyr216 phosphorylation, and elevated β‐catenin levels compared to oeVec‐YTN16 cells. However, these differences were no longer observed when cells were cultured with CKO CAFs' conditioned medium (Figure 6J).

To further validate these findings, we intraperitoneally injected oeVec‐YTN16 or oeGRIA2‐YTN16 cells into WT and CKO mice (Figure 6K). WT mice inoculated with oeGRIA2‐YTN16 cells displayed markedly increased metastatic burden relative to those receiving oeVec‐YTN16 cells (Figure 6L). However, in CKO mice, tumor metastasis was substantially attenuated, with no significant difference between the oeVec‐YTN16 and oeGRIA2‐YTN16 groups (Figure 6L). These results indicate that *Slc17a7* deficiency in CAFs effectively suppressed GRIA2‐mediated peritoneal metastasis.

Overall, these findings demonstrate that CAF‐derived glutamate in the peritoneal microenvironment enhances gastric cancer cell migration and metastasis through activation of GRIA2‐mediated Wnt/β‐catenin signaling.

### 2.7 Elevated GRIA2 Expression in Clinical Peritoneal Metastases Correlates with Wnt Activation and Poor Prognosis

**FIGURE 7:**
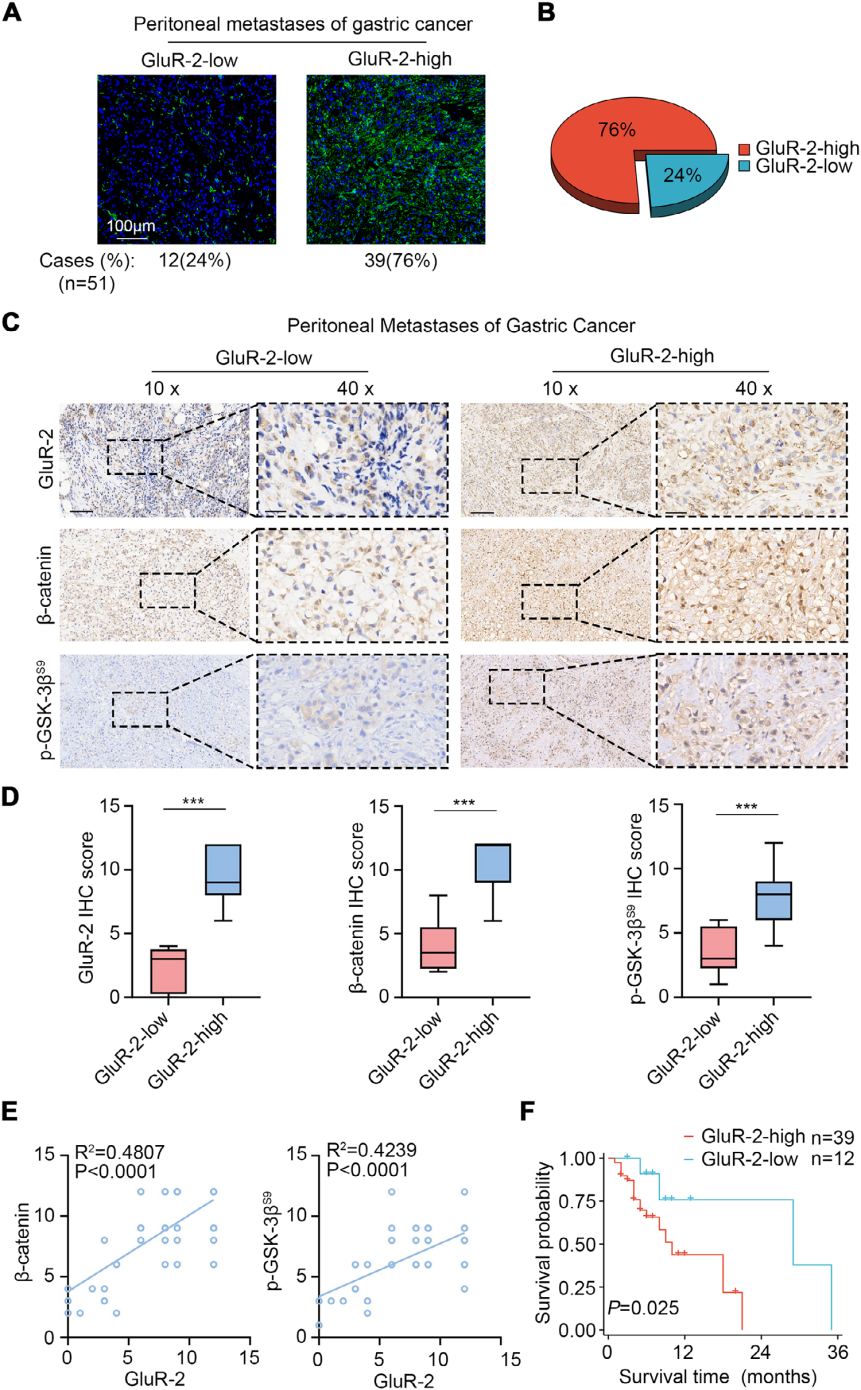
Elevated GRIA2 Expression in Clinical Peritoneal Metastases Correlates with Wnt Activation and Poor Prognosis. (A) Representative immunofluorescence images of GRIA2 expression in peritoneal metastases. (B) Pie chart showing the distribution of GRIA2‐high and GRIA2‐low cases (n = 51). (C) Representative immunohistochemical images of GRIA2, β‐catenin, and p‐GSK‐3βS9 in peritoneal metastases with GRIA2‐high (n = 39) versus GRIA2‐low (n = 12) expression. Scale bars, 50 µm (10×) and 25 µm (40×). (D) Quantitative evaluation of immunohistochemical staining intensity from samples in (C). (E) Correlation analysis between GRIA2 expression levels and β‐catenin or p‐GSK‐3βS9 protein expression levels in peritoneal metastatic lesions from gastric cancer patients. (F) Kaplan‐Meier survival curves of gastric cancer patients with peritoneal metastasis stratified by GRIA2 expression (n = 51, Zhongshan Hospital, Fudan University). Data in D were analyzed by the Mann‐Whitney U test. ^***^ *p* < 0.001.

To evaluate the clinical relevance of GRIA2 expression in gastric cancer peritoneal metastasis, we conducted GRIA2 immunofluorescence staining in peritoneal metastatic tumor tissues obtained from 51 gastric cancer patients (Figure 7A). Interestingly, GRIA2 was highly expressed in 76% of gastric cancer peritoneal metastatic lesions (Figure 7B). Immunohistochemical analysis revealed that peritoneal metastatic lesions with high GRIA2 expression exhibited elevated β‐catenin and phosphorylated GSK‐3β (Ser9) levels (Figure 7C,D). GRIA2 expression was positively correlated with both β‐catenin (R^2^ = 0.4807, p < 0.0001) and p‐GSK‐3β Ser9 (R^2^ = 0.4239, p < 0.0001) levels (Figure 7E). These results suggest that GRIA2, β‐catenin, and p‐GSK‐3β (Ser9) are co‐elevated in gastric cancer peritoneal metastases. We then analyzed whether GRIA2 expression in peritoneal metastatic lesions correlates with patient survival. In the Zhongshan Hospital cohort of gastric cancer patients with peritoneal metastasis (n = 51), patients with high GRIA2 expression demonstrated significantly shorter survival compared to those with low GRIA2 expression (Figure 7F).

These clinical data demonstrate that GRIA2 expression in gastric cancer peritoneal metastases correlates with Wnt/β‐catenin pathway activation and is associated with poor prognosis in gastric cancer patients with peritoneal metastasis.

## 3 Discussion

**FIGURE 8:**
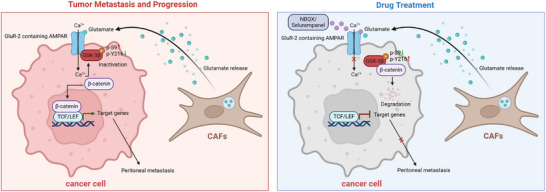
Schematic Model of GRIA2‐Mediated Peritoneal Metastasis in Gastric Cancer. CAFs secrete glutamate via SLC17A7, which binds to and activates GRIA2‐containing AMPA receptors on tumor cells. This triggers calcium influx and enhances the GRIA2‐GSK‐3β interaction, inhibiting GSK‐3β kinase activity and stabilizing β‐catenin, thereby activating Wnt signaling and driving peritoneal metastasis. AMPA receptor antagonists (NBQX and Selurampanel) block this signaling axis, promoting β‐catenin degradation and suppressing peritoneal metastasis. *Note: GRIA2* (gene) encodes the GluR‐2 protein.

In the present study, we performed an in vivo CRISPR/Cas9 loss‐of‐function screen and uncovered a role for GRIA2 in gastric cancer peritoneal metastasis. Our data delineate a signaling cascade in which CAF‐secreted glutamate engages AMPA receptors on tumor cells, leading to Ca^2^^+^‐dependent inactivation of GSK‐3β and downstream β‐catenin accumulation. Pharmacological interruption of this axis attenuated peritoneal spread in preclinical models (Figure 8).

GRIA2 has been classically characterized as a key mediator of excitatory neurotransmission in the central nervous system, with the A‐to‐I RNA editing event at the Q/R site and its regulation of Ca^2^^+^ permeability representing well‐established topics in neurobiology [34, 35]. Evidence linking ionotropic glutamate receptors, especially AMPA receptors, to tumor progression has largely come from tumors of neural origin. For instance, in glioblastoma, studies have shown that AMPAR‐mediated Ca^2^^+^ influx promotes tumor cell migration and proliferation to drive disease progression [36, 37]. However, functional evidence in epithelial malignancies remains limited. Our work provides direct evidence linking GRIA2 to gastric cancer peritoneal metastasis, extending the functional relevance of GRIA2 from neuronal disorders to epithelial tumor biology.

GSK‐3β serves as a critical component of the canonical Wnt/β‐catenin destruction complex, phosphorylating β‐catenin to promote its degradation and thereby regulating stem cell properties, proliferation, and migration [38, 39, 40]. Through IP‐MS, molecular dynamics simulations, and mutagenesis, we demonstrate that GRIA2 physically interacts with GSK‐3β, with R715 as a key binding residue and glutamate enhancing this interaction. Unedited GRIA2‐mediated calcium influx regulates GSK‐3β activity by altering phosphorylation at Ser9 and Tyr216. This is mechanistically compatible with a prior report showing that Ca^2^^+^ displaces Mg^2^^+^ at the GSK‐3β active site to inhibit catalysis [26]. This mechanism represents a previously unrecognized mode of β‐catenin activation in gastric cancer, and was further supported by bidirectional pharmacological modulation of GSK‐3β (Figure 4E; Figure S4D). Specifically, the GSK‐3β inhibitor LiCl rescued β‐catenin protein expression in GRIA2‐depleted cells, whereas the GSK‐3β activator DIF‐3 attenuated β‐catenin accumulation in GRIA2‐overexpressing cells. Mechanistically, LiCl inhibits GSK‐3β activity through direct competition with the essential cofactor Mg^2^^+^ at the metal‐binding site, thereby disrupting kinase catalytic function [41, 42, 43]. In GRIA2‐knockout cells, LiCl treatment suppressed GSK‐3β‐mediated phosphorylation of β‐catenin, thereby stabilizing β‐catenin protein levels. Conversely, DIF‐3 activates GSK‐3β through a dual mechanism: promoting dephosphorylation of the inhibitory Ser9 residue while enhancing phosphorylation of the activating Tyr216 residue [44]. In GRIA2‐overexpressing cells, DIF‐3 treatment enhanced GSK‐3β activity, thereby restoring β‐catenin phosphorylation and promoting its degradation [44, 45].

The relationship between ionotropic glutamate receptors and GSK‐3β activity has been documented in both physiological and pathological contexts. In neuronal systems, NMDA receptor activation modulates GSK‐3β phosphorylation status through a PKC‐dependent mechanism, increasing inhibitory Ser9 phosphorylation while reducing activating Tyr216 phosphorylation [46]. In the cancer setting, Ishiuchi and colleagues demonstrated that calcium‐permeable AMPA receptors promote glioblastoma cell proliferation and migration, and subsequently showed that this effect is mediated by calcium‐dependent phosphorylation of Akt at Ser473 [36, 37]. Activated Akt is known to phosphorylate GSK‐3β at Ser9, leading to its inhibition [47]. These observations suggest a potential indirect regulatory link between AMPA receptor‐mediated calcium influx and GSK‐3β suppression. Nevertheless, whether AMPA receptors can directly modulate GSK‐3β activity in epithelial malignancies has not been explored. Our study fills this gap by demonstrating that GRIA2 physically associates with GSK‐3β and suppresses its kinase activity in a calcium‐dependent manner.

Glutamate is a key mediator of synaptic plasticity, learning, and memory in the central nervous system [48]. Beyond its conventional neurobiological functions, glutamate also acts as a paracrine metabolite within the tumor microenvironment, where it exerts pleiotropic effects involving the modulation of immune cell differentiation and effector functions [49], preservation of redox homeostasis in malignant cells [50], and dysregulation of key signaling pathways [51]. Fibroblasts contribute significantly to cancer progression by releasing growth factors, cytokines, and metabolites, while also reshaping the extracellular matrix and promoting immunosuppression [52, 53]. Our findings demonstrate that cancer‐associated fibroblasts (CAFs) serve as the predominant source of glutamate in the gastric cancer peritoneal microenvironment. Through this paracrine supply of glutamate, CAFs establish a signaling axis that supports the metastatic behavior of gastric cancer cells.

NBQX and Selurampanel are competitive antagonists primarily targeting AMPA receptors, originally developed for neurological disorders including epilepsy [54, 55, 56]. Among these, Selurampanel has been tested in small clinical trials and shown a reasonably acceptable safety profile [54, 57]. Our investigation expands the potential therapeutic application of AMPA antagonists to gastric cancer peritoneal metastasis. Through evaluation using in vitro assays, patient‐derived organoids, and cell line‐derived and PDOX mouse models, we observed that NBQX and Selurampanel suppress metastasis‐associated phenotypes in vitro and inhibit peritoneal metastasis in vivo. Importantly, the differential efficacy of these agents in patient‐derived organoids with high versus low GRIA2 expression, as well as in mouse models, suggests that GRIA2 expression levels may serve as a predictive biomarker for precision therapy. Although NBQX and Selurampanel yielded encouraging preclinical results, their clinical development has focused on neurological disorders. Translation to oncological applications will require addressing challenges, including dose optimization, pharmacokinetics, potential neurological side effects, and safety profiles in cancer patients.

## 4 Conclusion

We define a GRIA2–Ca^2^^+^–GSK‐3β–β‐catenin signaling axis that drives gastric cancer peritoneal dissemination and identify AMPA receptor blockade as a potential therapeutic strategy, with GRIA2 serving as both a therapeutic target and a prognostic biomarker.

## 5 Experimental Section

### 5.1 Human Specimens

Paraffin‐embedded and fresh peritoneal metastatic tissue specimens were obtained from gastric cancer patients who underwent surgical treatment at Zhongshan Hospital, Fudan University (Shanghai, China). Paraffin‐embedded tissues were used for immunohistochemistry and immunofluorescence staining, while fresh tissues were processed for single‐cell RNA sequencing, flow cytometry analysis, and patient‐derived organoid (PDO) establishment. The clinicopathological characteristics of these patients are summarized in Supplementary Tables S10–S12. Written informed consent was obtained from all patients prior to specimen collection. This study was approved by the Ethics Committee of Zhongshan Hospital (Fudan University, Shanghai, China).

### 5.2 Cell Culture

Human gastric cancer cell lines (MKN74, MKN45, AGS, HGC‐27) were obtained from Procell Life Science & Technology (Wuhan, China) and cultured in RPMI‐1640 medium (Gibco). HMrSV5 cells were purchased from Yaji Biotech (Shanghai, China) and maintained in DMEM (Gibco). All media were supplemented with 10% fetal bovine serum (Gibco) and 1% penicillin‐streptomycin (Gibco). The YTN16 transplantable gastric cancer cell line, derived from C57BL/6 mice, was generously provided by the laboratory of Dr. Sachiyo Nomura (The University of Tokyo, Japan). YTN16 cells were maintained in Advanced DMEM (Gibco) with GlutaMAX (Gibco), 10% fetal bovine serum, and 1% penicillin‐streptomycin, cultured on plastic dishes pre‐coated with Type I collagen (Liver‐Biotech). Of note, standard RPMI‐1640 (Gibco) contains L‐glutamine (∼2 mm) and L‐glutamic acid (∼136 µm), and standard Advanced DMEM (Gibco) contains L‐glutamic acid (∼100 µm). Cells cultured in these media are therefore exposed to basal levels of glutamine and glutamate. For glutamate dose‐response experiments, customized glutamine‐ and glutamate‐free RPMI‐1640 or Advanced DMEM (Meilunbio) supplemented with 10% dialyzed FBS (YOBIBIO) and 2 mm GlutaMAX (Gibco) was used. GlutaMAX was used as a stable glutamine substitute that does not spontaneously degrade into glutamate in the culture medium. Exogenous L‐glutamate (MedChemExpress) was added at indicated concentrations (0, 50, or 100 µm). NBQX (MCE) and Selurampanel (MCE) were dissolved in dimethyl sulfoxide (DMSO) to prepare stock solutions. For all experiments, compounds were diluted in culture medium to achieve the indicated final concentrations, with a final DMSO concentration of 0.1% (v/v). Vehicle control groups received an equivalent concentration of DMSO. All cell lines were maintained at 37°C in a 5% CO_2_ incubator. Cell authenticity was verified by suppliers, and routine mycoplasma testing confirmed negative status.

### 5.3 Mice

All mice were housed in ventilated cages with hardwood chip substrate under environmentally controlled conditions with alternating 12‐h light and dark phases. Animals received a standard laboratory diet and sterile drinking water ad libitum. All experimental procedures were approved by the Institutional Animal Care and Use Committee of Zhongshan Hospital (Fudan University, Shanghai, China). *Slc17a7^+/+^/Fsp1‐CreER^T2^/Rosa26^LSL‐tdTomato^* mice and *Slc17a7^fl/fl^/Fsp1‐CreER^T2^/Rosa26^LSL‐tdTomato^* mice were generated by breeding *Slc17a7^fl/+^* mice with *Fsp1‐CreER^T2^*, which harbor a tamoxifen‐inducible improved Cre recombinase (*CreER^T2^*) driven by the *Fsp1* gene promoter, and with *Rosa26^LSL‐tdTomato^* mice (Cyagen). This system was utilized to specifically isolate fibroblasts that express tdTomato. Tamoxifen (MCE) was administered via intraperitoneal injection at a dose of 120 mg/kg daily for five consecutive days. Fourteen days later, tumor cells were injected intraperitoneally. Five days post‐inoculation, tamoxifen was administered again at 120 mg/kg daily for five consecutive days. Tumor burden was monitored by bioluminescence imaging (IVIS) at the indicated time points. At the experimental endpoint, mice were euthanized, and tissues were collected for analysis. A comprehensive summary of all in vivo mouse experiments is provided in Supplementary Table S13.

### 5.4 Genome‐Wide CRISPR Screening

To identify drivers of peritoneal metastasis in gastric cancer, we performed a genome‐wide CRISPR/Cas9 knockout screen using MKN74 cells. Approximately 1.6 × 10^8^ MKN74 cells were transduced with the human GeCKO v2 library (123,411 sgRNAs) at an MOI of 0.3. After puromycin selection and expansion, 1 × 10^8^ cells were collected as the pre‐injection control sample with greater than 500‐fold library coverage. The in vivo screen was conducted with 36 nude mice total, which were randomly divided into 3 replicate pools (12 mice per pool). Each mouse received 1 × 10^7^ transduced cells via intraperitoneal injection, achieving greater than 500‐fold coverage per replicate pool. All visible peritoneal metastatic lesions from the 12 mice within each replicate pool were harvested at the experimental endpoint (approximately 2.4 g tissue per pool) and pooled together for genomic DNA extraction. Genomic DNA was isolated using a salt‐precipitation method as previously described [27], yielding approximately 1,000 µg per replicate pool. The sgRNA cassettes were PCR‐amplified and sequenced on a NovaSeq X Plus platform (Illumina). sgRNA enrichment and depletion were analyzed using the MAGeCK‐RRA algorithm, comparing 3 in vivo replicate pools versus 3 pre‐injection control samples. Candidate genes were identified based on consistent sgRNA depletion across all 3 replicate pools, with the following criteria: p < 0.01, log_2_ fold‐change ≤ ‐2, and at least 3 sgRNAs showing consistent depletion.

### 5.5 CRISPR‐Cas9‐Mediated Gene Knockout

Gene knockout cell lines were established employing CRISPR‐Cas9 genome editing technology following previously published protocols. sgRNA sequences directed against GRIA2 were cloned into the lentiCRISPR v2 vector (Addgene). Lentiviral particles were produced through co‐transfection of HEK293T cells with the lentiCRISPR v2 construct alongside packaging plasmids pMD2.G (Addgene) and psPAX2 (Addgene). Gastric cancer cell lines MKN74 and MKN45 were subsequently infected with lentiviral supernatants harboring either non‐targeting control sgRNAs or gene‐specific sgRNAs. Following viral transduction, cells underwent selection with puromycin (2 µg/mL) for 72 h to eliminate non‐transduced cells. To obtain monoclonal knockout populations, surviving cells were subjected to limiting dilution by seeding into 96‐well plates at approximately one cell per well. Individual clones were expanded over 2–3 weeks, and successful gene ablation was validated through Western blot analysis. A non‐targeting control sgRNA from the GeCKO v2 library was used as the negative control. The gene‐specific sgRNA sequences are provided in Supplementary Table S1.

### 5.6 RNA Sequencing

Total RNA was extracted from MKN74 cells (sgGRIA2 and sgControl, n = 3 per group) using TRIzol reagent. RNA quality was verified using Bioanalyzer 2100 (RIN > 7.0). Paired‐end sequencing (150 bp) was performed on an Illumina NovaSeq 6000 platform (LC‐Bio Technologies, Hangzhou, China). Reads were aligned to the human reference genome (GRCh38) using HISAT2, and gene expression was quantified as FPKM using StringTie. Differential expression analysis was performed using DESeq2. Gene Ontology (GO) enrichment analysis was performed using the OmicStudio tools at https://www.omicstudio.cn/tool. To ensure statistical reliability and biological specificity, GO terms with gene set sizes between 15 and 300 were retained for analysis, excluding both underpowered small gene sets and overly broad terms lacking interpretive value. The Z‐score was calculated to indicate the overall direction of regulation for each enriched GO term, with negative Z‐scores representing predominant downregulation and positive Z‐scores indicating upregulation. GO terms with Z‐score < 0 were selected to identify biological processes downregulated upon GRIA2 depletion. Gene Set Enrichment Analysis (GSEA) was conducted using GSEA software with the KEGG pathway gene sets from the Molecular Signatures Database. The pathways with |NES|>1, NOM.pval < 0.05, and FDR.qval < 0.25 were considered significantly enriched.

### 5.7 RNA Interference‐Mediated Gene Silencing

Transient knockdown of β‐catenin expression was achieved using small interfering RNA (siRNA) technology. Cancer cells were reverse‐transfected with siRNA duplexes (GeneMeditech, Shanghai, China) at a final concentration of 20 nm utilizing Lipofectamine 3000 transfection reagent (Invitrogen) according to the manufacturer's protocol. Cells were harvested 48–72 h post‐transfection for downstream analyses. All siRNA sequences utilized in this study are detailed in Supplementary Table S2.

### 5.8 Plasmid Constructs and Transfection

Mouse Gria2, human GRIA2 mutants (R715A and Q607R), and human β‐catenin S33Y mutant were commercially synthesized (Unibio, China) and cloned into expression vectors with an N‐terminal Flag epitope tag. For stable GRIA2‐overexpressing YTN16 cells, lentiviral particles were produced by co‐transfecting HEK293T cells with pLVX‐Flag‐Gria2 (or empty vector), psPAX2 (Addgene), and pMD2.G (Addgene). YTN16 cells were transduced and selected with puromycin (2 µg/mL) for 7–10 days. Single‐cell clones were validated by Western blot. For transient transfection experiments, cells were transfected using Lipofectamine 3000 (Invitrogen) and harvested 48 h post‐transfection.

### 5.9 RNA Extraction and Quantitative PCR

Total RNA was extracted from cells and tissue samples using TRIzol reagent (Thermo Fisher Scientific) following the manufacturer's protocol. RNA was reverse transcribed using the PrimeScript RT reagent Kit (TaKaRa). Real‐time PCR was performed using SYBR Premix Ex Taq (TaKaRa). Primer sequences are listed in Supplementary Table S3. Relative mRNA expression was calculated using the comparative cycle threshold (2^−ΔΔCT) method.

### 5.10 Immunoblotting

Cellular lysates were prepared in RIPA buffer supplemented with PMSF (100:1 ratio). Protein concentrations were determined using the BCA Protein Assay Kit (Epizyme Biotechnology). Proteins (10–20 µg) underwent SDS‐PAGE separation and transfer to PVDF membranes. After blocking in 5% BSA‐TBST for 1 h, membranes were probed with primary antibodies overnight at 4°C, followed by three 10‐minute TBST washes. HRP‐conjugated secondary antibodies were applied for 1 h at room temperature. Following additional washing, chemiluminescence detection utilized ECL substrate (Sparkjade Biotechnology). Antibodies: anti‐β‐catenin (Proteintech, 66379‐1‐Ig), anti‐β‐Actin (Proteintech, 66009‐1‐Ig), anti‐GluR‐2 (ABclonal, A11316), anti‐GSK‐3β (CST, 12456), anti‐VGluT1 (Abcam, ab227805), anti‐DDDDK tag (Flag) (Abcam, ab205606), anti‐phospho‐GSK‐3β‐S389 (Proteintech, 14850‐1‐AP), anti‐phospho‐GSK‐3β‐Ser9 (Proteintech, 67558‐1‐Ig), anti‐phospho‐GSK3A/B‐Tyr279/216 (Proteintech, 29125‐1‐AP), HRP‐goat‐anti‐mouse (Proteintech, SA00001‐1), HRP‐goat‐anti‐rabbit (Proteintech, SA00001‐2).

### 5.11 Migration and Invasion Assays

Cell migration and invasion were evaluated using Transwell chambers (8 µm pore size). For invasion assays, the upper chamber membranes were pre‐coated with Matrigel (Corning). Cells were resuspended in 200 µL serum‐free medium and seeded into the upper chambers. The lower chambers were filled with 600 µL medium containing 10% FBS as a chemoattractant. After incubation for 24 h (migration) or 48 h (invasion) at 37°C, non‐migrated cells on the upper surface were removed with cotton swabs. Cells that migrated to the lower surface were fixed with 4% paraformaldehyde for 15 min, stained with 0.1% crystal violet for 20 min, and photographed under a light microscope. Cells were counted from five random fields per insert.

### 5.12 Generation of Luciferase‐Expressing Cell Lines and PDOs

All gastric cancer cell lines and PDOs used for in vivo experiments were transduced with lentivirus encoding firefly luciferase (Luc2) under the EF‐1α promoter. For cell lines (MKN74, MKN45, YTN16), cells were transduced with 8 µg/mL polybrene, selected with puromycin, and monoclonal lines were established by limiting dilution in 96‐well plates. For PDOs, organoids were dissociated into single cells using TrypLE Express and transduced with lentivirus in the presence of 8 µg/mL polybrene and 10 µm Y‐27632 to prevent dissociation‐induced anoikis. After embedding in Matrigel and recovery culture, puromycin‐selected organoids were dissociated and sparsely seeded to establish clonal lines. All clones were screened by IVIS imaging with D‐luciferin, and only those with stable and uniform bioluminescent signals were used for experiments.

### 5.13 Tissue Dissociation and Single‐Cell Suspension Preparation

Freshly resected peritoneal metastatic tumor tissues from gastric cancer patients were processed promptly after surgical removal. Tissues were mechanically minced into approximately 2–4 mm pieces and enzymatically dissociated using the Human Tumor Dissociation Kit (Miltenyi Biotec) according to the manufacturer's instructions. Briefly, tumor fragments were transferred into gentleMACS C Tubes containing the enzyme mix and dissociated using a gentleMACS Octo Dissociator with Heaters. The resulting cell suspensions were filtered through 70‐µm MACS SmartStrainers (Miltenyi Biotec) and washed with RPMI‐1640. Red blood cells were lysed using 1× Red Blood Cell Lysis Solution (Miltenyi Biotec). Cell viability was assessed by trypan blue exclusion, and only samples with viability > 80% were used for downstream analysis.

### 5.14 Fluorescence‐Activated Cell Sorting (FACS)

Cell sorting was performed using a BD FACSAria III cell sorter. Single‐cell suspensions were stained with fluorophore‐conjugated antibodies (listed in Table S5). The gating strategy for each cell population was as follows: epithelial cells (Viability Dye^−^CD45^−^CD31^−^EpCAM^+^), endothelial cells (Viability Dye^−^CD45^−^CD31^+^), CAFs (Viability Dye^−^CD45^−^EpCAM^−^CD31^−^PDGFRα^+^), neutrophils (Viability Dye^−^CD45^+^ CD11b^+^CD66b^+^CD15^+^CD14^−^), and dendritic cells (Viability Dye^−^CD45^+^ Lin^−^HLA‐DR^+^; Lin: CD3/CD14/CD19/ CD56). For the transgenic mice, tdTomato‐positive fibroblasts were sorted. Dead cells were excluded by Fixable Viability Dye eFluor 780.

### 5.15 Glutamate Detection

Sorted viable cells were cultured in customized glutamine‐ and glutamate‐free RPMI‐1640 (Meilunbio) supplemented with 10% dialyzed FBS (YOBIBIO) and 2 mm GlutaMAX (Gibco) for 8 h. Cell viability was reassessed at the end of incubation using trypan blue exclusion, and only samples with viability > 80% were included for analysis. Supernatants were collected and analyzed for extracellular glutamate levels using a fluorometric glutamate assay kit (Cell Biolabs, STA‐674) following the manufacturer's instructions. Glutamate levels were measured with a microplate reader (Thermo Fisher, Varioskan LUX) at Ex/Em 530–570/590‐600 nm. Results were normalized to cell number.

### 5.16 Calcium Influx Measurement

Cells seeded in 12‐well plates were loaded with 4 µm Fluo‐4 AM (Thermo Fisher) in calcium‐free buffer for 30 min at 37°C in the dark. After washing, cells were incubated in calcium‐free Hanks' Balanced Salt Solution (HBSS). Calcium influx was initiated by adding 2.5 mm CaCl_2_, and fluorescence was continuously recorded using an IX83 fluorescence microscope (Olympus). Fluorescence intensity was quantified using ImageJ software. Changes were calculated as maximal fluorescence (Fmax) relative to baseline (F0).

### 5.17 Co‐Immunoprecipitation and Mass Spectrometry

To identify GRIA2‐interacting proteins, MKN74 cells were transfected with Flag‐tagged GRIA2 using Lipofectamine 3000 (Thermo Fisher). After 48 h, cell lysis was performed in immunoprecipitation lysis buffer on ice for 45 min. Lysates were immunoprecipitated with Anti‐FLAG M2 Magnetic Beads (Sigma–Aldrich) for 4 h at 4°C, followed by three consecutive washes with 1×PBS. Precipitated proteins were separated by SDS‐PAGE and subjected to in‐gel trypsin digestion. The resulting peptides were analyzed by LC‐MS/MS.

### 5.18 GSK‐3β Activity Assay

GSK‐3β was immunoprecipitated from cell lysates using an anti‐GSK‐3β antibody. The kinase activity of immunoprecipitated GSK‐3β was subsequently assessed using a bioluminescent ADP‐Glo kinase assay system (Promega) according to the manufacturer's instructions. Luminescence signals were quantified on a GloMax 96 Microplate Luminometer (Promega), and relative kinase activity was normalized to control samples.

### 5.19 Sphere Formation Assay

Cells (1×10^4^ per well of MKN74, MKN45, or YTN16) were inoculated in ultralow‐attachment plates with sphere medium: DMEM/F12 supplemented with 2 mM L‐glutamine, 100 U/mL penicillin‐streptomycin, 20 ng/mL recombinant EGF (R&D Systems), 10 ng/mL recombinant bFGF (R&D Systems), and 1×B27 supplement (Gibco). Spheres were cultured for 14 days before analysis. For glutamate dose‐response sphere formation assays, customized glutamine‐ and glutamate‐free DMEM/F12 (Meilunbio) was used instead, with 2 mM GlutaMAX (Gibco) replacing L‐glutamine and all other supplements remaining the same.

### 5.20 Tumor‐Mesothelial Adhesion Assay

Human peritoneal mesothelial cells (HMrSV5) or mouse peritoneal mesothelial cells were seeded in 96‐well plates and cultured overnight until reaching confluency. MKN74, MKN45, or YTN16 cells were labeled with 15 µM calcein AM (Thermo Fisher) for 30 min at 37°C and then added (5×10^4^ cells per well) to the human or mouse peritoneal mesothelial monolayer for 3 h of co‐culture. Non‐adherent cells were removed by three washes with 200 µL growth medium, and adherent tumor cells were observed and photographed under an inverted microscope.

### 5.21 Immunohistochemistry and Immunofluorescence Analysis

Tissue specimens from gastric cancer peritoneal metastases were processed as formalin‐fixed, paraffin‐embedded (FFPE) sections. Following deparaffinization by baking at 60°C for 6 h and xylene treatment, sections underwent rehydration via a descending ethanol gradient. Heat‐induced epitope retrieval (HIER) was conducted in citrate buffer (pH 6.0) using microwave irradiation. Endogenous peroxidase was quenched by exposure to 3% hydrogen peroxide for 10 min. Sections were then blocked and incubated with primary antibodies overnight at 4°C. Detection was achieved using Primary Antibody Amplifier Quanto, followed by HRP Polymer Quanto (Thermo Fisher Scientific). Chromogenic visualization was performed with 3,3'‐diaminobenzidine (DAB), and nuclear counterstaining was accomplished with hematoxylin. Immunohistochemical staining was semi‐quantitatively evaluated using the Immunoreactive Score (IRS). The percentage of positive cells (PP) was scored as: 0 (no positive cells), 1 (<10%), 2 (10%–50%), 3 (51%–80%), or 4 (>80%). Staining intensity (SI) was scored as: 0 (negative), 1 (weak), 2 (moderate), or 3 (strong). The final IRS was calculated as PP × SI (range 0–12). Two experienced pathologists independently scored all samples under blinded conditions. Samples with IRS ≥6 were classified as high expression, while those with IRS <6 were classified as low expression.

For immunofluorescence, antigen retrieval was accomplished using citrate buffer (pH 6.0). Following membrane permeabilization with 0.5% Triton X‐100, tissues were probed with primary antibodies and subsequently labeled with Alexa Fluor 488‐tagged anti‐rabbit secondary antibodies (Abcam, ab150077). Nuclear demarcation was achieved with DAPI staining. Fluorescent signals were acquired and quantified utilizing the HALO digital pathology platform (Indica Labs).

### 5.22 Patient‐Derived Organoid Culture

Clinically obtained tumor specimens were placed in preservation solution (BioGenous) and washed with DPBS containing 1% penicillin‐streptomycin. Tumor parenchyma was trimmed and digested in the primary tumor digestion solution (BioGenous). Digestion was terminated with fetal bovine serum, followed by centrifugation. Pellets were treated with erythrocyte lysis buffer (BioGenous), washed with DPBS, resuspended in matrix gel (Mogengel Bio), and plated in 24‐well plates (Sangon Biotech). Organoids were maintained in gastric cancer complete medium (Mogengel Bio).

### 5.23 Mouse Model of Peritoneal Dissemination

Male nude mice and C57BL/6J mice (aged 5–6 weeks) were used to establish peritoneal dissemination models. Luciferase‐labeled MKN74 or MKN45 cells with GRIA2 knockout, luciferase‐labeled YTN16 cells overexpressing GRIA2, and their corresponding control cell lines were cultured until reaching the exponential growth phase. Following trypsinization and enumeration, cell suspensions were prepared at concentrations of 1×10^6^ or 1×10^7^ cells per injection in PBS, respectively. Tumor cells were inoculated intraperitoneally on day 0. For bioluminescence imaging, D‐Luciferin potassium salt was administered intraperitoneally 10–15 min before imaging. Tumor progression was periodically monitored through bioluminescence imaging using an IVIS Spectrum imaging system (PerkinElmer).

### 5.24 Patient‐Derived Organoid Xenograft (PDOX) Model

Sex‐matched NKG mice (6 weeks old, Cyagen Biosciences) were used for PDOX establishment. PDOs (1×10^6^ cells) suspended in 50 µL Matrigel (Corning) were injected intraperitoneally. Tumor burden was monitored by bioluminescence imaging as described above.

### 5.25 Single‐Cell RNA Sequencing

Gastric cancer peritoneal metastasis specimens were collected from patients, rinsed in ice‐cold PBS, and dissociated using a tumor dissociation kit (Miltenyi Biotec) to generate single‐cell suspensions. GEMs (Gel Beads‐in‐emulsion) were generated by combining barcoded Single Cell 5' Gel Beads, a Master Mix with cells, and Partitioning Oil on a Chromium controller (10x Genomics). Barcoded full‐length cDNA was generated through reverse transcription and amplified via PCR. The 5' gene expression libraries were constructed and sequenced on the Illumina NovaSeq 6000 platform with a PE150 strategy. Raw sequencing data were processed using the 10X Genomics Cell Ranger pipeline (version 3.1.0). Low‐quality cells containing fewer than 200 detected genes or greater than 20% mitochondrial gene expression were excluded. Doublets coexpressing multiple cell‐type‐specific markers were further removed. The filtered data were subsequently analyzed using the Seurat pipeline, with Uniform Manifold Approximation and Projection (UMAP) applied for dimensionality reduction and visualization. To evaluate SLC17A6 and SLC17A7 expression heterogeneity across cell types and samples, the single‐cell dataset was stratified by sample identity into individual subsets. The DotPlot function in the Seurat package was employed to calculate the average expression level and expression percentage of target genes within each cell cluster per sample. The arithmetic mean of these metrics across all independent samples was computed to generate robust summary statistics.

### 5.26 Survival Analysis

Kaplan‐Meier curves for overall survival were generated using GraphPad Prism, stratifying patients by GRIA2 protein expression levels in tissue samples. The prognostic significance of candidate gene expression in gastric cancer was assessed through the Kaplan–Meier plotter platform (https://kmplot.com/analysis/).

### 5.27 Statistical Analysis

Prior to statistical analysis, the normality of data distribution was assessed using the Shapiro‐Wilk test, with p > 0.05 indicating normal distribution. For normally distributed data, parametric tests were applied: unpaired Student's t‐test for two‐group comparisons, one‐way analysis of variance (ANOVA) for comparisons among three or more groups, and two‐way ANOVA for experiments involving two independent variables. For data not meeting the normality assumption, non‐parametric alternatives were employed: the Mann‐Whitney U test for two‐group comparisons and the Kruskal‐Wallis test for multiple‐group comparisons. For one‐way ANOVA with significant results (p < 0.05), Tukey's Honest Significant Difference (HSD) test was used for pairwise comparisons among all groups, and Dunnett's test was applied for comparisons of multiple treatment groups against a single control. For two‐way ANOVA, Bonferroni correction was applied for post hoc multiple comparisons. Detailed statistical information is provided in Supplementary Table S4. P < 0.05 was considered statistically significant. Data are presented as means ± standard deviation (SD). Statistical analyses were performed using GraphPad Prism 10.

## Author Contributions

Conception and design: X.W., Y.R., and H.L.; Genome‐wide CRISPR screening: Y.W., and C.J.; Investigation: J.S., X.Y., and W.J.; Acquisition of patient specimens: H.S., and X.L.; Paper drafting and revising: J.S., X.Y., X.W., and J.Z.; Provision of YTN16 cell line: M.Y., T.T., and S.N. All authors approved the final version of the paper.

## Ethics Statement

The study design received approval from the Institutional Ethics Committee of Zhongshan Hospital (Fudan University, Shanghai, China), and informed consent was obtained from all study participants.

## Conflicts of Interest

The authors declare no conflicts of interest.

## Supporting information




**Supporting File 1**: advs74711‐sup‐0001‐FigureS1‐S6.docx.

**Supporting File 2**: advs74711‐sup‐0002‐TableS2.docx.

**Supporting File 3**: advs74711‐sup‐0003‐TableS2.xlsx.

## Data Availability

The data that support the findings of this study are available on request from the corresponding author. The data are not publicly available due to privacy or ethical restrictions.
