# Supplementary material for: In Vivo CRISPR Screening Identifies the Glutamate Receptor GRIA2 as Promoting Peritoneal Metastasis of Gastric Cancer via Calcium‐Dependent β‐Catenin Activation
Source: Adv Sci (Weinh). 2026 Mar 10;13(28):e21746. doi: 10.1002/advs.202521746 (PMC13185854; doi:10.1002/advs.202521746)
Supplement: Supplementary file 1 — Supporting File 1: advs74711‐sup‐0001‐FigureS1‐S6.docx. [file ADVS-13-e21746-s002.docx]

**Supplementary Figures and Figure Legends**

**Supplementary Figure S1**

**
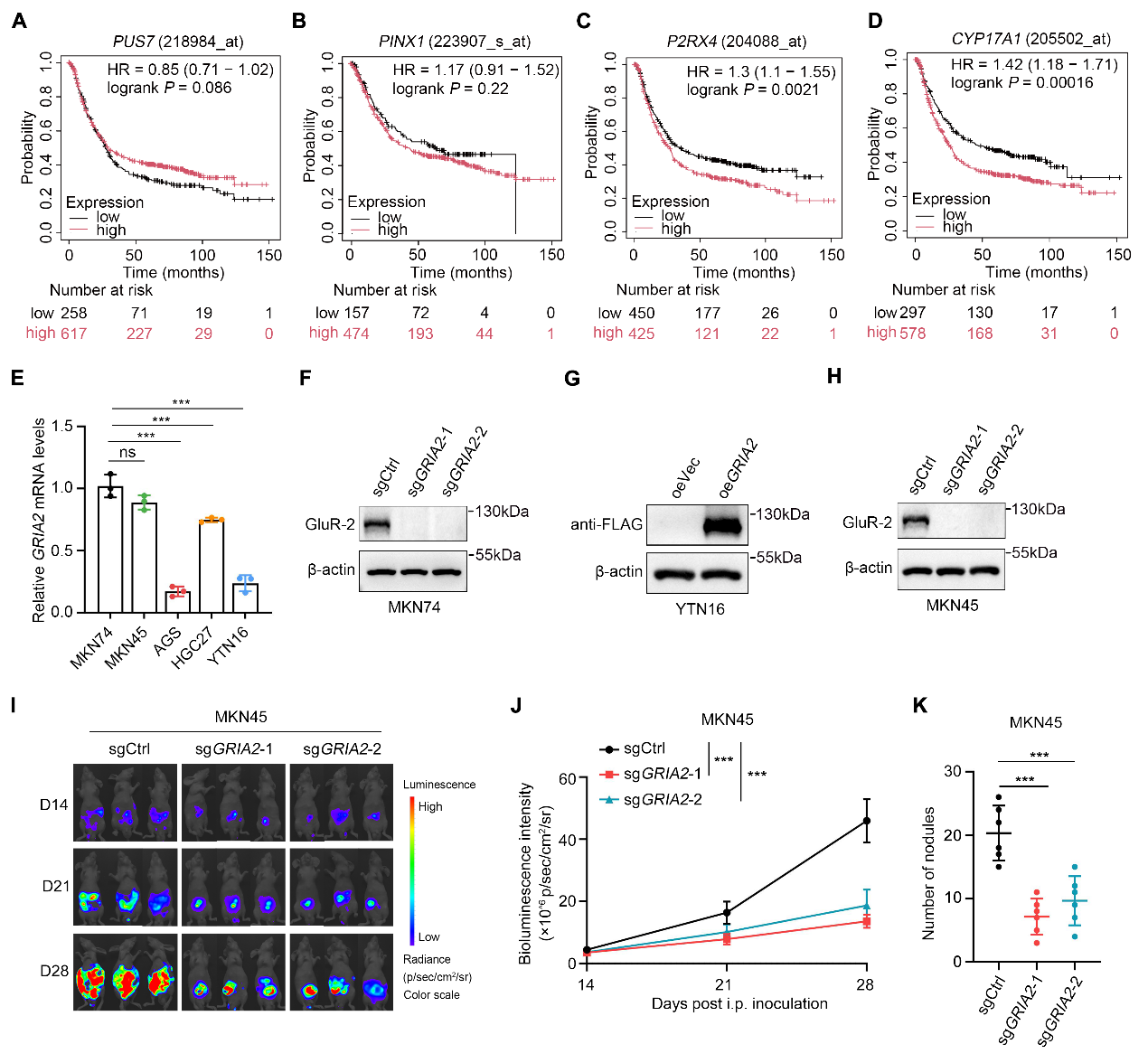
**

**Figure S1.** Genome-wide CRISPR screening identifies GRIA2 as a critical driver of gastric cancer peritoneal metastasis. A-D) Kaplan-Meier survival curves depicting the relationship between expression levels of PUS7 (A), PINX1 (B), P2RX4 (C), and CYP17A1 (D) and overall survival in gastric cancer patients. E) Relative mRNA expression levels of GRIA2 across multiple gastric cancer cell lines. F) Western blot confirmation of GluR-2 knockout efficiency in MKN74 cells. G) Western blot analysis using anti-FLAG antibody to verify overexpression of FLAG-GRIA2 in YTN16 cells. H) Confirmation of GluR-2 knockout in MKN45 cells was performed by immunoblotting. I) IVIS imaging of tumor progression in mice following intraperitoneal injection of luciferase-labeled MKN45 cells (sgCtrl versus sgGRIA2) (n = 6 per group). J) Quantification of bioluminescence signal intensity from (I). K) Quantification of intraperitoneal metastatic nodule numbers from the experiment in (I). One-way ANOVA was used for E and K. Two-way ANOVA was used for J. Data are shown as the mean ± s.d. ****P* < 0.001; NS, not significant.

**Supplementary Figure S2**

**
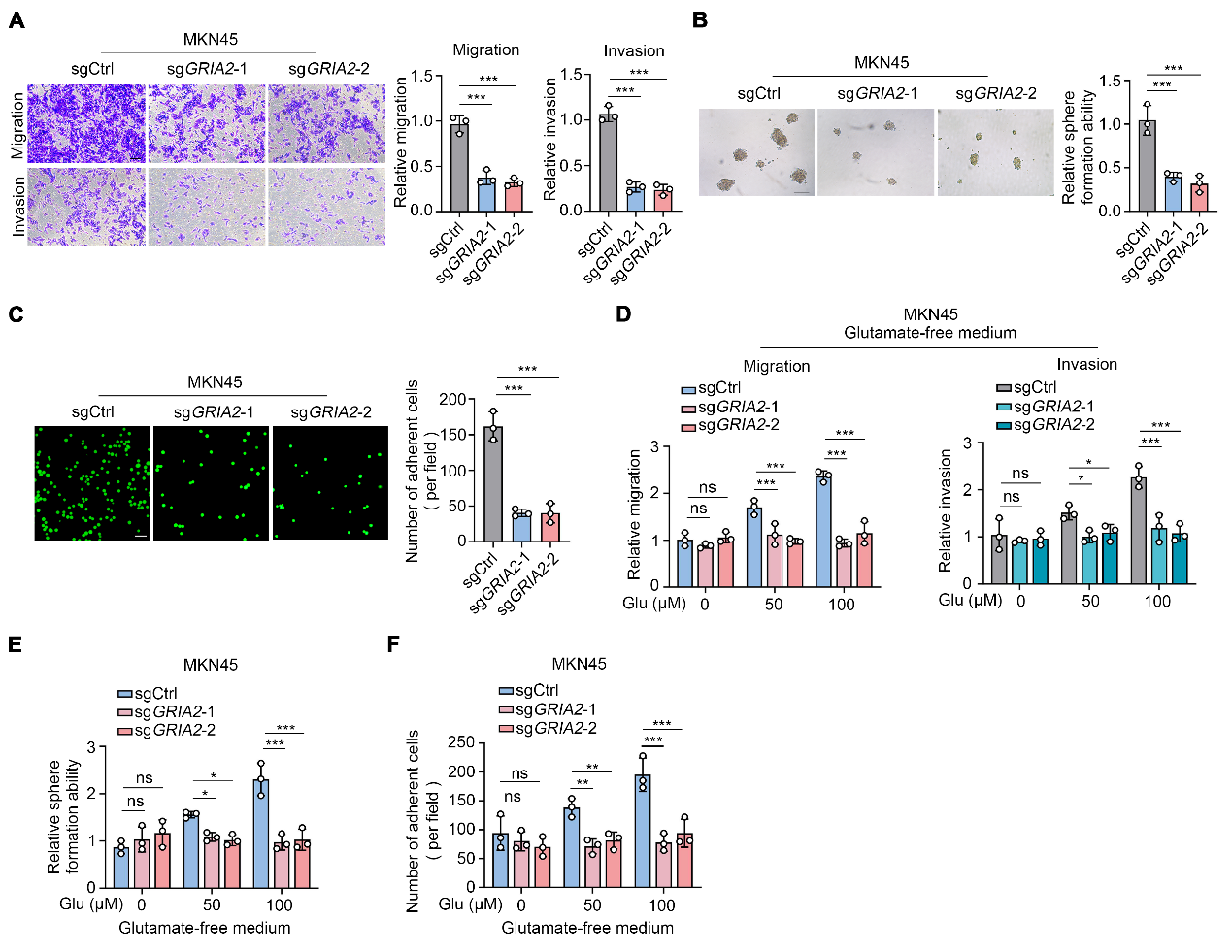
**

**Figure S2.** GRIA2 promotes gastric cancer cell migration, invasion, stemness, and peritoneal adhesion in a glutamate-dependent manner. A-C) Transwell assays examining the effects of GRIA2 knockout on MKN45 cell migration and invasion (A), sphere formation (B), and adhesion to peritoneal mesothelial cells (C) (n = 3). Scale bars: 50 μm (A, C) and 150 μm (B). A–C were performed in standard culture medium containing basal levels of glutamine and glutamate. D-F) Effects of GRIA2 depletion on MKN45 cell migration and invasion (D), sphere formation (E), and mesothelial cell adhesion (F) under varying glutamate concentrations (n = 3). D–F were performed in customized glutamine- and glutamate-free medium supplemented with GlutaMAX, with exogenous L-glutamate added at the indicated concentrations. One-way ANOVA was applied to A–C. D–F were analyzed by two-way ANOVA. Data are shown as the mean ± s.d. **P* < 0.05, ***P* < 0.01, ****P* < 0.001; NS indicates non-significance.

**Supplementary Figure S3**

**
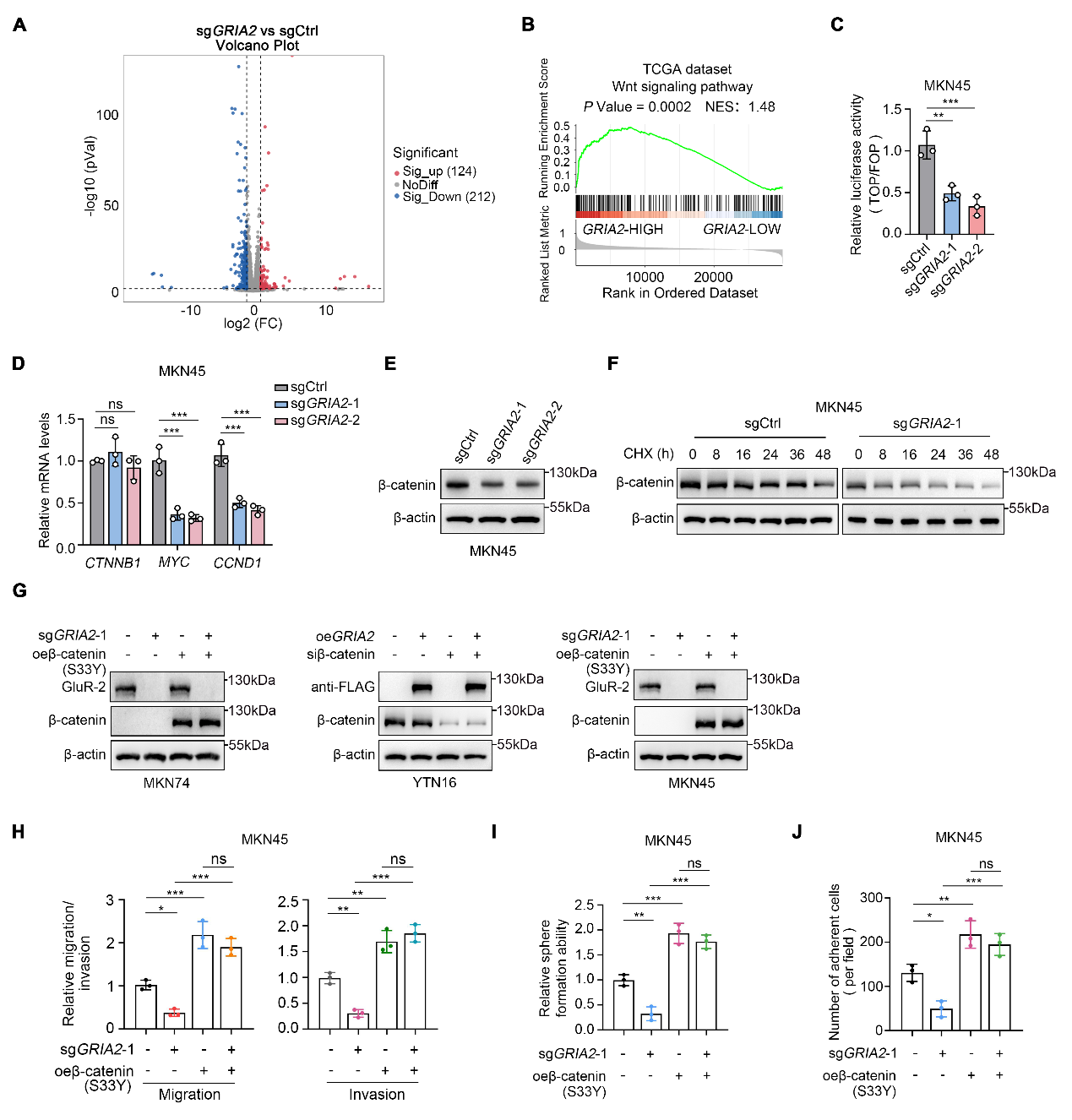
**

**Figure S3.** GRIA2 drives gastric cancer metastatic phenotypes through Wnt/β-catenin pathway activation. A) Volcano plot displaying differentially expressed genes between GRIA2-knockout (sgGRIA2) and control (sgCtrl) MKN74 cells. B) GSEA based on TCGA gastric cancer dataset demonstrating positive correlation between GRIA2 expression and Wnt signaling gene signatures. C) Impact of GRIA2 knockout on TOP/FOP flash reporter activity in MKN45 cells (n = 3). D) Alterations in *CTNNB1*, *MYC*, and *CCND1* mRNA levels in MKN45 cells following GRIA2 depletion (n = 3). E) Immunoblot detection of β-catenin protein expression in GRIA2-depleted MKN45 cells. F) CHX chase experiment evaluating the effect of GRIA2 knockout on β-catenin protein stability in MKN45 cells. G) Western blot analysis of GluR-2 and β-catenin protein levels in MKN74 and MKN45 cells (sgCtrl or sgGRIA2) with or without mutant β-catenin (S33Y) expression, and in YTN16 cells (vector or GRIA2 overexpression) with or without β-catenin siRNA transfection. H-J) Rescue experiments showing that mutant β-catenin (S33Y) expression restores migration and invasion (H), sphere formation (I), and mesothelial adhesion (J) in GRIA2-depleted MKN45 cells (n = 3). All experiments were performed in standard culture medium containing basal levels of glutamine and glutamate. C, H, I, and J were analyzed using one-way ANOVA. D was analyzed by two-way ANOVA. Data are shown as the mean ± s.d. **P* < 0.05, ***P* < 0.01, ****P* < 0.001; NS, not significant.

**Supplementary Figure S4**

**
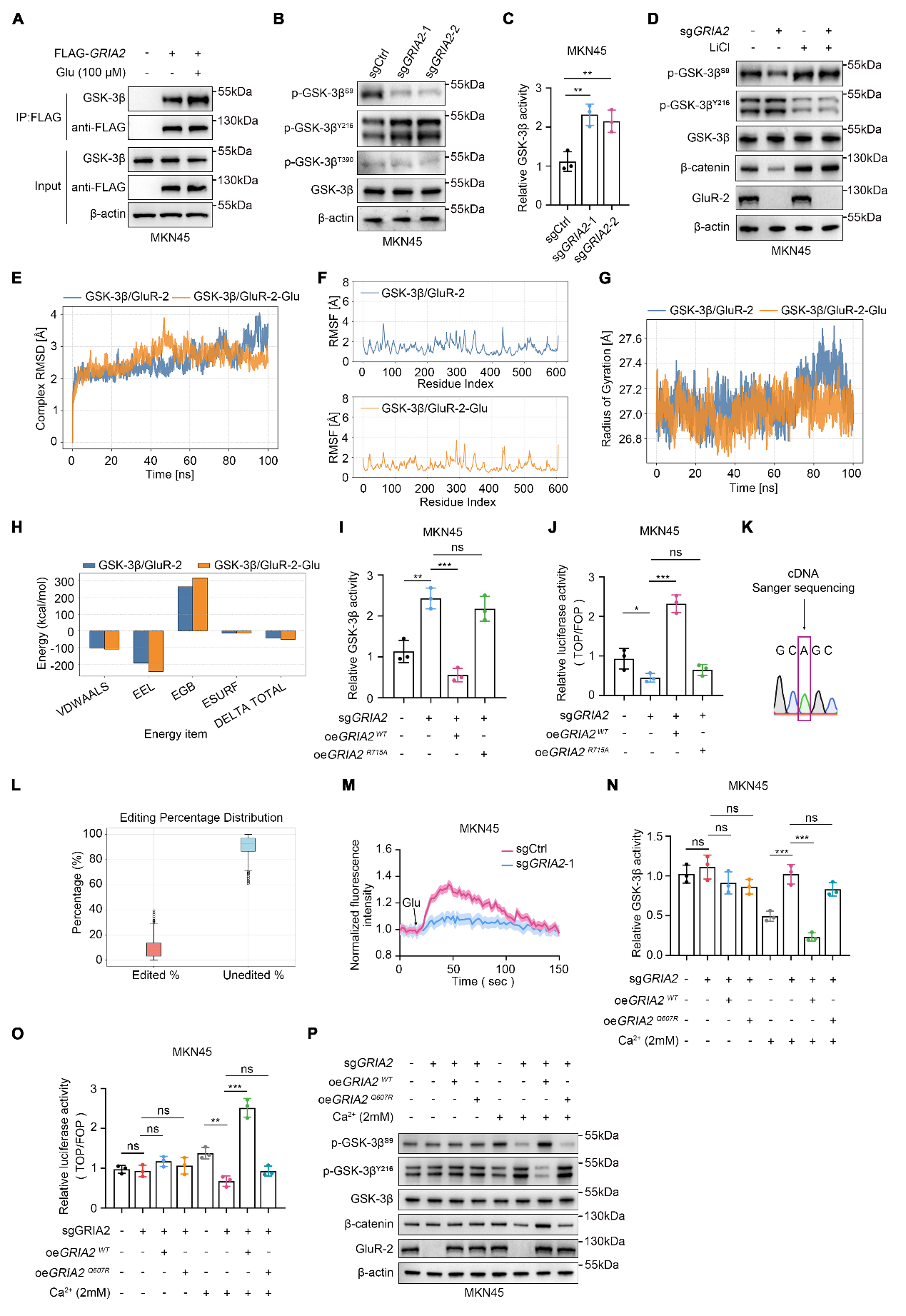
**

**Figure S4.** GRIA2 physically interacts with GSK-3β and inhibits its kinase activity via calcium influx to stabilize β-catenin. A) Co-IP analysis of the interaction between GRIA2 and GSK-3β in MKN45 cells, with or without glutamate stimulation. B) Impact of GRIA2 knockout on GSK-3β phosphorylation levels and total GSK-3β in MKN45 cells. C) Assessment of GSK-3β enzymatic activity alterations in GRIA2-depleted MKN45 cells (n = 3). D) Immunoblot analysis of GSK-3β phosphorylation and β-catenin expression. E) RMSD analysis of the GRIA2–GSK-3β complex from molecular dynamics simulations with or without glutamate binding. F) RMSF analysis of the GRIA2–GSK-3β complex calculated from simulation trajectories. G) Radius of gyration of the GRIA2–GSK-3β complex over simulation time. H) Binding energy calculation using MM/GBSA method. VDWAALS: van der Waals energy; EEL: electrostatic energy; EGB: electrostatic contribution to solvation; ESURF: non-polar contribution to solvation; DELTA TOTAL: binding free energy. I) GSK-3β activity following reconstitution with wild-type GRIA2 or R715A mutant in GRIA2-knockout MKN45 cells (n = 3). J) Detection of the impact of overexpressing wild-type or R715A mutant on TOP/FOP flash activity in MKN45 cells (n = 3). K) Sanger sequencing confirming Q607R editing status in gastric cancer cells. L) Box plot showing the distribution of GRIA2 Q607R editing levels in the TCGA-STAD dataset. M) Measurement of intracellular calcium influx in control and GRIA2-knockout MKN45 cells. N-P) Effects of wild-type or Q607R mutant on GSK-3β activity (N) (n = 3), TOP/FOP activity (O) (n = 3), and related protein expression (P) in MKN45 cells with or without calcium. Experiments in A–D, I, and J were performed in standard culture medium containing basal levels of glutamine and glutamate. For N–P, customized calcium-free RPMI-1640 containing basal levels of glutamine and glutamate was used, with or without CaCl₂ (2 mM) supplementation. C, I, J, N and O were analyzed using one-way ANOVA. Data are shown as the mean ± s.d. **P* < 0.05, ***P* < 0.01, ****P* < 0.001; NS, not significant.

**Supplementary Figure S5**

**
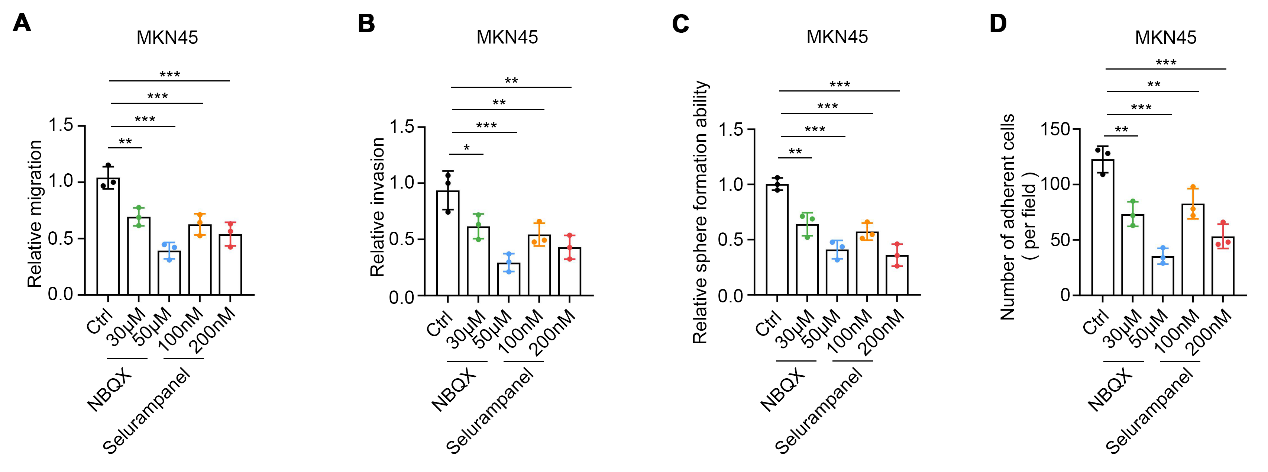
**

**Figure S5.** AMPA receptor antagonists suppress gastric cancer peritoneal metastasis in preclinical models. A) Effects of NBQX or Selurampanel at indicated concentrations on MKN45 cell migration (n = 3). B) Effects of NBQX or Selurampanel at indicated concentrations on MKN45 cell invasion (n = 3). C) Effects of NBQX or Selurampanel at indicated concentrations on MKN45 cell sphere formation (n = 3). D) Effects of NBQX or Selurampanel at indicated concentrations on MKN45 cell adhesion to peritoneal mesothelial cells (n = 3). Experiments in A–D were performed in standard culture medium containing basal levels of glutamine and glutamate. P values in A–D were analyzed by one-way ANOVA. Data are shown as the mean ± s.d. **P* < 0.05, ***P* < 0.01, ****P* < 0.001.

**Supplementary Figure S6**

**
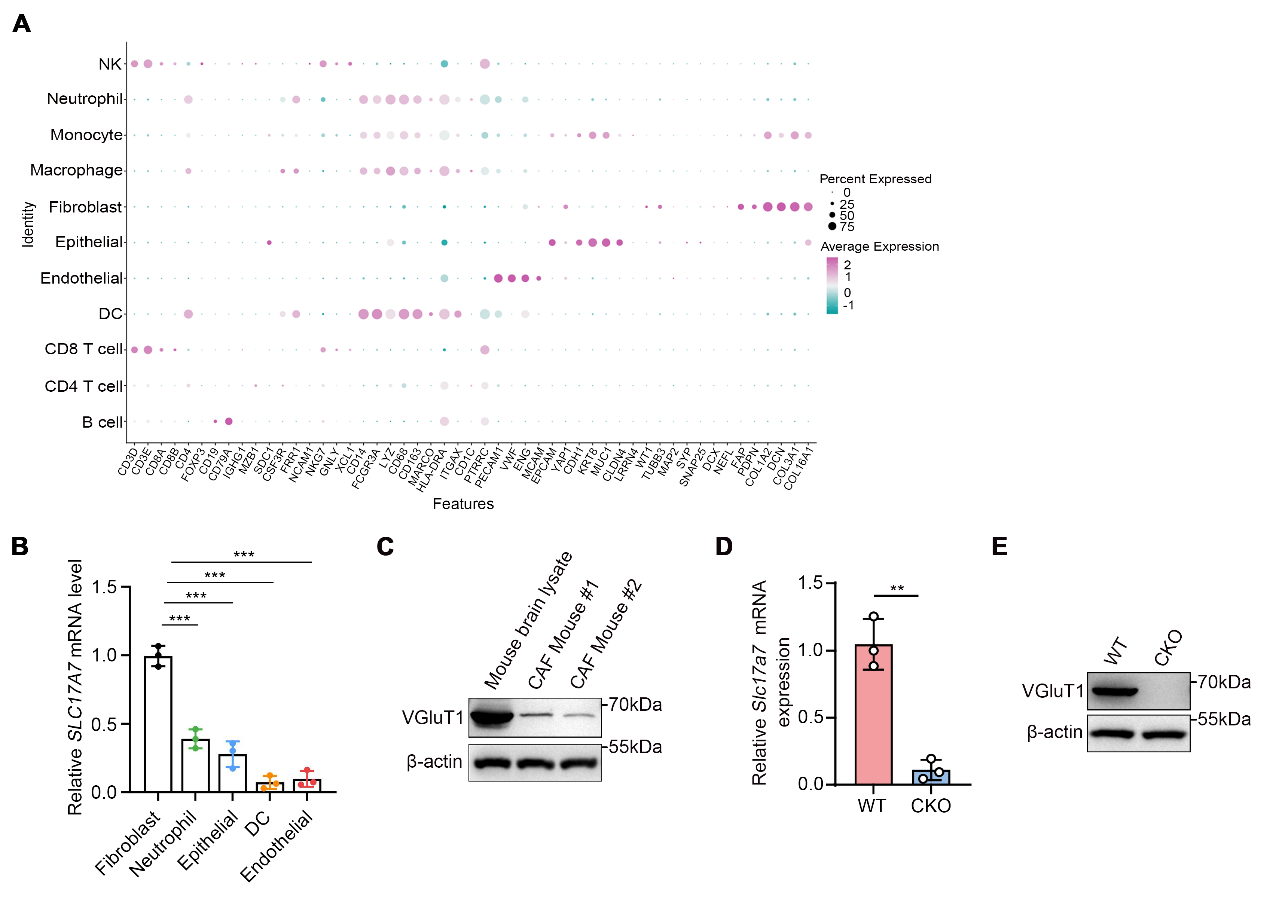
**

**Figure S6.** Cancer-Associated Fibroblast-Derived Glutamate Drives GRIA2-Mediated Peritoneal Metastasis. A) Dot plot showing the expression profiles of marker genes utilized for defining different cell types. B) Relative mRNA expression of SLC17A7 in different cell types determined by RT-qPCR. C) Western blot analysis of VGluT1 protein expression in CAFs isolated from peritoneal metastases. Mouse brain lysate was used as a positive control. D) Real-time PCR analysis of *Slc17a7* mRNA expression in sorted CAFs. E) Western blot analysis of VGluT1 protein. B was analyzed by one-way ANOVA. D was analyzed by unpaired t-test. Data are shown as the mean ± s.d. ***P* < 0.01, ****P* < 0.001; NS, not significant.
