# Supplementary material for: In Vivo CRISPR Screening Identifies the Glutamate Receptor GRIA2 as Promoting Peritoneal Metastasis of Gastric Cancer via Calcium‐Dependent β‐Catenin Activation
Source: Adv Sci (Weinh). 2026 Mar 10;13(28):e21746. doi: 10.1002/advs.202521746 (PMC13185854; doi:10.1002/advs.202521746)
Supplement: Supplementary file 2 — Supporting File 2: advs74711‐sup‐0002‐TableS2.docx. [file ADVS-13-e21746-s003.docx]

**Supplementary Table S1**

sgRNAs for CRISPR knockout

| **Gene** | **sgRNA sequence** |
| --- | --- |
| GRIA2-sgRNA1 | TGTCAGTCTGAACTCCGAAG |
| GRIA2-sgRNA2 | GACACCCCACATCGACAATT |

**Supplementary Table S2**

siRNA sequences used in this study

| **Name** | **Sequence (5'-3')** |
| --- | --- |
| Non-targeting control | UUCUCCGAACGUGUCACGUTT |
| β-catenin-si #1 | GCAAGCUCAUUGAUACCUATT |
| β-catenin-si #2 | GGUAGAUACCAGCCCACAUTT |

**Supplementary Table S3**

Primer sequences used for real-time PCR

| Gene | Forward/Reverse | Primer sequence |
| --- | --- | --- |
| CTNNB1 | Forward | 5′- CACAAGCAGAGTGCTGAAGGTG -3′ |
|  | Reverse | 5′- GATTCCTGAGAGTCCAAAGACAG -3′ |
| MYC | Forward | 5′- CCTGGTGCTCCATGAGGAGAC -3′ |
|  | Reverse | 5′- CAGACTCTGACCTTTTGCCAGG -3′ |
| CCND1 | Forward | 5′- TCTACACCGACAACTCCATCCG -3′ |
|  | Reverse | 5′- TCTGGCATTTTGGAGAGGAAGTG -3′ |
| GRIA2 | Forward | 5′- CCTGGATTCCAAAGGCTATGGC -3′ |
|  | Reverse | 5′- GTCTAAGACGCCTTGCTCACTG -3′ |
| ACTB | Forward | 5′- CACCATTGGCAATGAGCGGTTC -3′ |
|  | Reverse | 5′- AGGTCTTTGCGGATGTCCACGT -3′ |
| Ctnnb1 | Forward | 5′- GTTCGCCTTCATTATGGACTGCC -3′ |
|  | Reverse | 5′- ATAGCACCCTGTTCCCGCAAAG -3′ |
| Myc | Forward | 5′- TCGCTGCTGTCCTCCGAGTCC -3′ |
|  | Reverse | 5′- GGTTTGCCTCTTCTCCACAGAC -3′ |
| Ccnd1 | Forward | 5′- GCAGAAGGAGATTGTGCCATCC -3′ |
|  | Reverse | 5′- AGGAAGCGGTCCAGGTAGTTCA -3′ |
| Gria2 | Forward | 5′- TTCCTTGGGTGCCTTTATGCGG -3′ |
|  | Reverse | 5′- CACCATCCTCTCTACAGTCAGG -3′ |
| Actb | Forward | 5′- CATTGCTGACAGGATGCAGAAGG -3′ |
|  | Reverse | 5′- TGCTGGAAGGTGGACAGTGAGG -3′ |

**Supplementary Table S4**

Detailed Statistical Analysis Results

| **Figure** | **Comparison** | **Test** | **Statistic** | **df** | **P-value** |
| --- | --- | --- | --- | --- | --- |
| Fig 1F | Group | Two-way ANOVA | F = 81.14 | (2, 45) | <0.0001 |
|  | sgCtrl vs sgGRIA2-1 (Day 28) | Bonferroni post hoc | - | - | <0.0001 |
|  | sgCtrl vs sgGRIA2-2 (Day 28) | Bonferroni post hoc | - | - | <0.0001 |
| Fig 1G | Group | One-way ANOVA | F = 24.22 | (2, 15) | <0.0001 |
|  | sgCtrl vs sgGRIA2-1 | Tukey's HSD | - | - | 0.0001 |
|  | sgCtrl vs sgGRIA2-2 | Tukey's HSD | - | - | <0.0001 |
| Fig 1I | Group | Two-way ANOVA | F = 57.13 | (1, 30) | <0.0001 |
|  | oeVec vs oeGRIA2 | Bonferroni post hoc | - | - | <0.0001 |
| Fig 1J | oeVec vs oeGRIA2 | Unpaired t-test | t=3.948 | 10 | 0.0027 |
| Fig S1E | Overall | One-way ANOVA | F = 126.1 | (4, 10) | <0.0001 |
|  | MKN74 vs MKN45 | Dunnett's test | - | - | 0.0685 |
|  | MKN74 vs AGS | Dunnett's test | - | - | <0.0001 |
|  | MKN74 vs HGC27 | Dunnett's test | - | - | 0.0008 |
|  | MKN74 vs YTN16 | Dunnett's test | - | - | <0.0001 |
| Fig S1J | Group | Two-way ANOVA | F = 86.88 | (2, 45) | <0.0001 |
|  | sgCtrl vs sgGRIA2-1 (Day 28) | Bonferroni post hoc | - | - | <0.0001 |
|  | sgCtrl vs sgGRIA2-2 (Day 28) | Bonferroni post hoc | - | - | <0.0001 |
| Fig S1K | Group | One-way ANOVA | F = 21.01 | (2, 15) | <0.0001 |
|  | sgCtrl vs sgGRIA2-1 | Tukey's HSD | - | - | <0.0001 |
|  | sgCtrl vs sgGRIA2-2 | Tukey's HSD | - | - | 0.0005 |
| Fig 2A (MKN74 migration) | Group | One-way ANOVA | F = 18.45 | (2, 6) | 0.0027 |
|  | sgCtrl vs sgGRIA2-1 | Dunnett's test | - | - | 0.0043 |
|  | sgCtrl vs sgGRIA2-2 | Dunnett's test | - | - | 0.0028 |
| Fig 2A (MKN74 invasion) | Group | One-way ANOVA | F = 45.52 | (2, 6) | 0.0002 |
|  | sgCtrl vs sgGRIA2-1 | Dunnett's test | - | - | 0.0002 |
|  | sgCtrl vs sgGRIA2-2 | Dunnett's test | - | - | 0.0005 |
| Fig 2A (YTN16 migration) | oeVec vs oeGRIA2 | Unpaired t-test | t=5.969 | 4 | 0.004 |
| Fig 2A (YTN16 invasion) | oeVec vs oeGRIA2 | Unpaired t-test | t=7.878 | 4 | 0.0014 |
| Fig S2A (MKN45 migration) | Group | One-way ANOVA | F = 67.29 | (2, 6) | <0.0001 |
|  | sgCtrl vs sgGRIA2-1 | Dunnett's test | - | - | 0.0001 |
|  | sgCtrl vs sgGRIA2-2 | Dunnett's test | - | - | <0.0001 |
| Fig S2A (MKN45 invasion) | Group | One-way ANOVA | F = 147.2 | (2, 6) | <0.0001 |
|  | sgCtrl vs sgGRIA2-1 | Dunnett's test | - | - | <0.0001 |
|  | sgCtrl vs sgGRIA2-2 | Dunnett's test | - | - | <0.0001 |
| Fig 2B (MKN74) | Group | One-way ANOVA | F = 30.38 | (2, 6) | 0.0007 |
|  | sgCtrl vs sgGRIA2-1 | Dunnett's test | - | - | 0.0011 |
|  | sgCtrl vs sgGRIA2-2 | Dunnett's test | - | - | 0.0008 |
| Fig 2B (YTN16) | oeVec vs oeGRIA2 | Unpaired t-test | t=3.937 | 4 | 0.017 |
| Fig S2B (MKN45) | Group | One-way ANOVA | F = 35.3 | (2, 6) | 0.0005 |
|  | sgCtrl vs sgGRIA2-1 | Dunnett's test | - | - | 0.0009 |
|  | sgCtrl vs sgGRIA2-2 | Dunnett's test | - | - | 0.0005 |
| Fig 2C (MKN74) | Group | One-way ANOVA | F = 41.48 | (2, 6) | 0.0003 |
|  | sgCtrl vs sgGRIA2-1 | Dunnett's test | - | - | 0.0003 |
|  | sgCtrl vs sgGRIA2-2 | Dunnett's test | - | - | 0.0007 |
| Fig 2C (YTN16) | oeVec vs oeGRIA2 | Unpaired t-test | t=8.904 | 4 | 0.0009 |
| Fig S2C (MKN45) | Group | One-way ANOVA | F = 72.05 | (2, 6) | <0.0001 |
|  | sgCtrl vs sgGRIA2-1 | Dunnett's test | - | - | <0.0001 |
|  | sgCtrl vs sgGRIA2-2 | Dunnett's test | - | - | <0.0001 |
| Fig 2D (MKN74 migration) | Group | Two-way ANOVA | F = 29.67 | (2, 18) | <0.0001 |
|  | sgCtrl vs sgGRIA2-1 (0 μM) | Bonferroni post hoc | - | - | >0.9999 |
|  | sgCtrl vs sgGRIA2-2 (0 μM) | Bonferroni post hoc | - | - | 0.9053 |
|  | sgCtrl vs sgGRIA2-1 (50 μM) | Bonferroni post hoc | - | - | 0.0069 |
|  | sgCtrl vs sgGRIA2-2 (50 μM) | Bonferroni post hoc | - | - | 0.0032 |
|  | sgCtrl vs sgGRIA2-1 (100 μM) | Bonferroni post hoc | - | - | <0.0001 |
|  | sgCtrl vs sgGRIA2-2 (100 μM) | Bonferroni post hoc | - | - | <0.0001 |
| Fig 2D (MKN74 invasion) | Group | Two-way ANOVA | F = 41.95 | (2, 18) | <0.0001 |
|  | sgCtrl vs sgGRIA2-1 (0 μM) | Bonferroni post hoc | - | - | >0.9999 |
|  | sgCtrl vs sgGRIA2-2 (0 μM) | Bonferroni post hoc | - | - | >0.9999 |
|  | sgCtrl vs sgGRIA2-1 (50 μM) | Bonferroni post hoc | - | - | 0.0125 |
|  | sgCtrl vs sgGRIA2-2 (50 μM) | Bonferroni post hoc | - | - | 0.0022 |
|  | sgCtrl vs sgGRIA2-1 (100 μM) | Bonferroni post hoc | - | - | <0.0001 |
|  | sgCtrl vs sgGRIA2-2 (100 μM) | Bonferroni post hoc | - | - | <0.0001 |
| Fig 2D (YTN16 migration) | Group | Two-way ANOVA | F = 57.51 | (1, 12) | <0.0001 |
|  | oeVec vs oeGRIA2 (0 μM) | Bonferroni post hoc | - | - | 0.3397 |
|  | oeVec vs oeGRIA2 (50 μM) | Bonferroni post hoc | - | - | 0.0001 |
|  | oeVec vs oeGRIA2 (100 μM) | Bonferroni post hoc | - | - | <0.0001 |
| Fig 2D (YTN16 invasion) | Group | Two-way ANOVA | F = 35.93 | (1, 12) | <0.0001 |
|  | oeVec vs oeGRIA2 (0 μM) | Bonferroni post hoc | - | - | 0.2580 |
|  | oeVec vs oeGRIA2 (50 μM) | Bonferroni post hoc | - | - | 0.0019 |
|  | oeVec vs oeGRIA2 (100 μM) | Bonferroni post hoc | - | - | <0.0001 |
| Fig S2D (MKN45 migration) | Group | Two-way ANOVA | F = 63.76 | (2, 18) | <0.0001 |
|  | sgCtrl vs sgGRIA2-1 (0 μM) | Bonferroni post hoc | - | - | 0.8368 |
|  | sgCtrl vs sgGRIA2-2 (0 μM) | Bonferroni post hoc | - | - | >0.9999 |
|  | sgCtrl vs sgGRIA2-1 (50 μM) | Bonferroni post hoc | - | - | 0.0004 |
|  | sgCtrl vs sgGRIA2-2 (50 μM) | Bonferroni post hoc | - | - | <0.0001 |
|  | sgCtrl vs sgGRIA2-1 (100 μM) | Bonferroni post hoc | - | - | <0.0001 |
|  | sgCtrl vs sgGRIA2-2 (100 μM) | Bonferroni post hoc | - | - | <0.0001 |
| Fig S2D (MKN45 invasion) | Group | Two-way ANOVA | F = 24.23 | (2, 18) | <0.0001 |
|  | sgCtrl vs sgGRIA2-1 (0 μM) | Bonferroni post hoc | - | - | >0.9999 |
|  | sgCtrl vs sgGRIA2-2 (0 μM) | Bonferroni post hoc | - | - | >0.9999 |
|  | sgCtrl vs sgGRIA2-1 (50 μM) | Bonferroni post hoc | - | - | 0.0188 |
|  | sgCtrl vs sgGRIA2-2 (50 μM) | Bonferroni post hoc | - | - | 0.0376 |
|  | sgCtrl vs sgGRIA2-1 (100 μM) | Bonferroni post hoc | - | - | <0.0001 |
|  | sgCtrl vs sgGRIA2-2 (100 μM) | Bonferroni post hoc | - | - | <0.0001 |
| Fig 2E (MKN74) | Group | Two-way ANOVA | F = 43.41 | (2, 18) | <0.0001 |
|  | sgCtrl vs sgGRIA2-1 (0 μM) | Bonferroni post hoc | - | - | >0.9999 |
|  | sgCtrl vs sgGRIA2-2 (0 μM) | Bonferroni post hoc | - | - | >0.9999 |
|  | sgCtrl vs sgGRIA2-1 (50 μM) | Bonferroni post hoc | - | - | 0.0064 |
|  | sgCtrl vs sgGRIA2-2 (50 μM) | Bonferroni post hoc | - | - | 0.0036 |
|  | sgCtrl vs sgGRIA2-1 (100 μM) | Bonferroni post hoc | - | - | <0.0001 |
|  | sgCtrl vs sgGRIA2-2 (100 μM) | Bonferroni post hoc | - | - | <0.0001 |
| Fig 2E (YTN16) | Group | Two-way ANOVA | F = 60.03 | (1, 12) | <0.0001 |
|  | oeVec vs oeGRIA2 (0 μM) | Bonferroni post hoc | - | - | 0.3978 |
|  | oeVec vs oeGRIA2 (50 μM) | Bonferroni post hoc | - | - | 0.0005 |
|  | oeVec vs oeGRIA2 (100 μM) | Bonferroni post hoc | - | - | <0.0001 |
| Fig S2E (MKN45) | Group | Two-way ANOVA | F = 19.34 | (2, 18) | <0.0001 |
|  | sgCtrl vs sgGRIA2-1 (0 μM) | Bonferroni post hoc | - | - | >0.9999 |
|  | sgCtrl vs sgGRIA2-2 (0 μM) | Bonferroni post hoc | - | - | 0.2807 |
|  | sgCtrl vs sgGRIA2-1 (50 μM) | Bonferroni post hoc | - | - | 0.0374 |
|  | sgCtrl vs sgGRIA2-2 (50 μM) | Bonferroni post hoc | - | - | 0.0154 |
|  | sgCtrl vs sgGRIA2-1 (100 μM) | Bonferroni post hoc | - | - | <0.0001 |
|  | sgCtrl vs sgGRIA2-2 (100 μM) | Bonferroni post hoc | - | - | <0.0001 |
| Fig 2F (MKN74) | Group | Two-way ANOVA | F = 19.85 | (2, 18) | <0.0001 |
|  | sgCtrl vs sgGRIA2-1 (0 μM) | Bonferroni post hoc | - | - | >0.9999 |
|  | sgCtrl vs sgGRIA2-2 (0 μM) | Bonferroni post hoc | - | - | >0.9999 |
|  | sgCtrl vs sgGRIA2-1 (50 μM) | Bonferroni post hoc | - | - | 0.0111 |
|  | sgCtrl vs sgGRIA2-2 (50 μM) | Bonferroni post hoc | - | - | 0.0174 |
|  | sgCtrl vs sgGRIA2-1 (100 μM) | Bonferroni post hoc | - | - | <0.0001 |
|  | sgCtrl vs sgGRIA2-2 (100 μM) | Bonferroni post hoc | - | - | <0.0001 |
| Fig 2F (YTN16) | Group | Two-way ANOVA | F = 38.44 | (1, 12) | <0.0001 |
|  | oeVec vs oeGRIA2 (0 μM) | Bonferroni post hoc | - | - | 0.7995 |
|  | oeVec vs oeGRIA2 (50 μM) | Bonferroni post hoc | - | - | 0.0028 |
|  | oeVec vs oeGRIA2 (100 μM) | Bonferroni post hoc | - | - | <0.0001 |
| Fig S2F (MKN45) | Group | Two-way ANOVA | F = 29.26 | (2, 18) | <0.0001 |
|  | sgCtrl vs sgGRIA2-1 (0 μM) | Bonferroni post hoc | - | - | >0.9999 |
|  | sgCtrl vs sgGRIA2-2 (0 μM) | Bonferroni post hoc | - | - | 0.5171 |
|  | sgCtrl vs sgGRIA2-1 (50 μM) | Bonferroni post hoc | - | - | 0.0023 |
|  | sgCtrl vs sgGRIA2-2 (50 μM) | Bonferroni post hoc | - | - | 0.0091 |
|  | sgCtrl vs sgGRIA2-1 (100 μM) | Bonferroni post hoc | - | - | <0.0001 |
|  | sgCtrl vs sgGRIA2-2 (100 μM) | Bonferroni post hoc | - | - | <0.0001 |
| Fig 3C (MKN74) | Group | One-way ANOVA | F = 33.86 | (2, 6) | 0.0005 |
|  | sgCtrl vs sgGRIA2-1 | Dunnett's test | - | - | 0.0007 |
|  | sgCtrl vs sgGRIA2-2 | Dunnett's test | - | - | 0.0007 |
| Fig 3C (YTN16) | oeVec vs oeGRIA2 | Unpaired t-test | t=8.853 | 4 | 0.0009 |
| Fig S3C (MKN45) | Group | One-way ANOVA | F = 27.29 | (2, 6) | 0.001 |
|  | sgCtrl vs sgGRIA2-1 | Dunnett's test | - | - | 0.0027 |
|  | sgCtrl vs sgGRIA2-2 | Dunnett's test | - | - | 0.0008 |
| Fig 3D (MKN74) | Group | Two-way ANOVA | F = 68.44 | (2, 18) | <0.0001 |
|  | sgCtrl vs sgGRIA2-1 (CTNNB1) | Bonferroni post hoc | - | - | 0.9989 |
|  | sgCtrl vs sgGRIA2-2 (CTNNB1) | Bonferroni post hoc | - | - | >0.9999 |
|  | sgCtrl vs sgGRIA2-1 (MYC) | Bonferroni post hoc | - | - | <0.0001 |
|  | sgCtrl vs sgGRIA2-2 (MYC) | Bonferroni post hoc | - | - | <0.0001 |
|  | sgCtrl vs sgGRIA2-1 (CCND1) | Bonferroni post hoc | - | - | <0.0001 |
|  | sgCtrl vs sgGRIA2-2 (CCND1) | Bonferroni post hoc | - | - | <0.0001 |
| Fig 3D (YTN16) | Group | Two-way ANOVA | F = 97.95 | (1, 12) | <0.0001 |
|  | oeVec vs oeGRIA2 (CTNNB1) | Bonferroni post hoc | - | - | 0.4963 |
|  | oeVec vs oeGRIA2 (Myc) | Bonferroni post hoc | - | - | <0.0001 |
|  | oeVec vs oeGRIA2 (CCND1) | Bonferroni post hoc | - | - | <0.0001 |
| Fig S3D (MKN45) | Group | Two-way ANOVA | F = 48.37 | (2, 18) | <0.0001 |
|  | sgCtrl vs sgGRIA2-1 (CTNNB1) | Bonferroni post hoc | - | - | 0.6678 |
|  | sgCtrl vs sgGRIA2-2 (CTNNB1) | Bonferroni post hoc | - | - | >0.9999 |
|  | sgCtrl vs sgGRIA2-1 (MYC) | Bonferroni post hoc | - | - | <0.0001 |
|  | sgCtrl vs sgGRIA2-2 (MYC) | Bonferroni post hoc | - | - | <0.0001 |
|  | sgCtrl vs sgGRIA2-1 (CCND1) | Bonferroni post hoc | - | - | <0.0001 |
|  | sgCtrl vs sgGRIA2-2 (CCND1) | Bonferroni post hoc | - | - | <0.0001 |
| Fig 3G (MKN74) (Migration) | Group | One-way ANOVA | F = 37.17 | (3, 8) | <0.0001 |
|  | ctrl vs sgGRIA2 | Tukey's HSD | - | - | 0.0318 |
|  | ctrl vs oeβ-cat(S33Y) | Tukey's HSD | - | - | 0.0023 |
|  | sgGRIA2 vs sgGRIA2+ oeβ-cat(S33Y) | Tukey's HSD | - | - | 0.0001 |
|  | oeβ-cat(S33Y) vs sgGRIA2+ oeβ-cat(S33Y) | Tukey's HSD | - | - | 0.8980 |
| Fig 3G (MKN74) (Invasion) | Group | One-way ANOVA | F = 32.75 | (3, 8) | <0.0001 |
|  | ctrl vs sgGRIA2 | Tukey's HSD | - | - | 0.0370 |
|  | ctrl vs oeβ-cat(S33Y) | Tukey's HSD | - | - | 0.0021 |
|  | sgGRIA2 vs sgGRIA2+ oeβ-cat(S33Y) | Tukey's HSD | - | - | 0.0004 |
|  | oeβ-cat(S33Y) vs sgGRIA2+ oeβ-cat(S33Y) | Tukey's HSD | - | - | 0.3278 |
| Fig 3G (YTN16) (Migration) | Group | One-way ANOVA | F = 76.8 | (3, 8) | <0.0001 |
|  | ctrl vs oeGRIA2 | Tukey's HSD | - | - | <0.0001 |
|  | ctrl vs siβ-cat | Tukey's HSD | - | - | 0.0195 |
|  | oeGRIA2 vs oeGRIA2+ siβ-cat | Tukey's HSD | - | - | <0.0001 |
|  | siβ-cat vs oeGRIA2+ siβ-cat | Tukey's HSD | - | - | 0.9797 |
| Fig 3G (YTN16) (Invasion) | Group | One-way ANOVA | F = 51.89 | (3, 8) | <0.0001 |
|  | ctrl vs oeGRIA2 | Tukey's HSD | - | - | 0.0005 |
|  | ctrl vs siβ-cat | Tukey's HSD | - | - | 0.0112 |
|  | oeGRIA2 vs oeGRIA2+ siβ-cat | Tukey's HSD | - | - | <0.0001 |
|  | siβ-cat vs oeGRIA2+ siβ-cat | Tukey's HSD | - | - | 0.6263 |
| Fig S3H (MKN45) (Migration) | Group | One-way ANOVA | F = 51.57 | (3, 8) | <0.0001 |
|  | ctrl vs sgGRIA2 | Tukey's HSD | - | - | 0.0176 |
|  | ctrl vs oeβ-cat(S33Y) | Tukey's HSD | - | - | 0.0004 |
|  | sgGRIA2 vs sgGRIA2+ oeβ-cat(S33Y) | Tukey's HSD | - | - | <0.0001 |
|  | oeβ-cat(S33Y) vs sgGRIA2+ oeβ-cat(S33Y) | Tukey's HSD | - | - | 0.3637 |
| Fig S3H (MKN45) (Invasion) | Group | One-way ANOVA | F = 66.45 | (3, 8) | <0.0001 |
|  | ctrl vs sgGRIA2 | Tukey's HSD | - | - | 0.0025 |
|  | ctrl vs oeβ-cat(S33Y) | Tukey's HSD | - | - | 0.0020 |
|  | sgGRIA2 vs sgGRIA2+ oeβ-cat(S33Y) | Tukey's HSD | - | - | <0.0001 |
|  | oeβ-cat(S33Y) vs sgGRIA2+ oeβ-cat(S33Y) | Tukey's HSD | - | - | 0.5900 |
| Fig 3H (MKN74) | Group | One-way ANOVA | F = 56.73 | (3, 8) | <0.0001 |
|  | ctrl vs sgGRIA2 | Tukey's HSD | - | - | 0.0298 |
|  | ctrl vs oeβ-cat(S33Y) | Tukey's HSD | - | - | 0.0002 |
|  | sgGRIA2 vs sgGRIA2+ oeβ-cat(S33Y) | Tukey's HSD | - | - | <0.0001 |
|  | oeβ-cat(S33Y) vs sgGRIA2+ oeβ-cat(S33Y) | Tukey's HSD | - | - | 0.0691 |
| Fig 3H (YTN16) | Group | One-way ANOVA | F = 378.2 | (3, 8) | <0.0001 |
|  | ctrl vs oeGRIA2 | Tukey's HSD | - | - | <0.0001 |
|  | ctrl vs siβ-cat | Tukey's HSD | - | - | <0.0001 |
|  | oeGRIA2 vs oeGRIA2+ siβ-cat | Tukey's HSD | - | - | <0.0001 |
|  | siβ-cat vs oeGRIA2+ siβ-cat | Tukey's HSD | - | - | 0.5541 |
| Fig S3I (MKN45) | Group | One-way ANOVA | F = 72.63 | (3, 8) | <0.0001 |
|  | ctrl vs sgGRIA2 | Tukey's HSD | - | - | 0.0027 |
|  | ctrl vs oeβ-cat(S33Y) | Tukey's HSD | - | - | 0.0003 |
|  | sgGRIA2 vs sgGRIA2+ oeβ-cat(S33Y) | Tukey's HSD | - | - | <0.0001 |
|  | oeβ-cat(S33Y) vs sgGRIA2+ oeβ-cat(S33Y) | Tukey's HSD | - | - | 0.5580 |
| Fig 3I (MKN74) | Group | One-way ANOVA | F = 32.99 | (3, 8) | <0.0001 |
|  | ctrl vs sgGRIA2 | Tukey's HSD | - | - | 0.0190 |
|  | ctrl vs oeβ-cat(S33Y) | Tukey's HSD | - | - | 0.0032 |
|  | sgGRIA2 vs sgGRIA2+ oeβ-cat(S33Y) | Tukey's HSD | - | - | 0.0004 |
|  | oeβ-cat(S33Y) vs sgGRIA2+ oeβ-cat(S33Y) | Tukey's HSD | - | - | 0.3299 |
| Fig 3I (YTN16) | Group | One-way ANOVA | F = 63.56 | (3, 8) | <0.0001 |
|  | ctrl vs oeGRIA2 | Tukey's HSD | - | - | <0.0001 |
|  | ctrl vs siβ-cat | Tukey's HSD | - | - | 0.0387 |
|  | oeGRIA2 vs oeGRIA2+ siβ-cat | Tukey's HSD | - | - | <0.0001 |
|  | siβ-cat vs oeGRIA2+ siβ-cat | Tukey's HSD | - | - | 0.6750 |
| Fig S3J (MKN45) | Group | One-way ANOVA | F = 29.86 | (3, 8) | 0.0001 |
|  | ctrl vs sgGRIA2 | Tukey's HSD | - | - | 0.0131 |
|  | ctrl vs oeβ-cat(S33Y) | Tukey's HSD | - | - | 0.0094 |
|  | sgGRIA2 vs sgGRIA2+ oeβ-cat(S33Y) | Tukey's HSD | - | - | 0.0003 |
|  | oeβ-cat(S33Y) vs sgGRIA2+ oeβ-cat(S33Y) | Tukey's HSD | - | - | 0.6658 |
| Fig 4D (MKN74) | Group | One-way ANOVA | F = 16.85 | (2, 6) | 0.0035 |
|  | sgCtrl vs sgGRIA2-1 | Dunnett's test | - | - | 0.0126 |
|  | sgCtrl vs sgGRIA2-2 | Dunnett's test | - | - | 0.0024 |
| Fig 4D (YTN16) | oeVec vs oeGRIA2 | Unpaired t-test | t=5.21 | 4 | 0.0065 |
| Fig S4C (MKN45) | Group | One-way ANOVA | F = 17.46 | (2, 6) | 0.0032 |
|  | sgCtrl vs sgGRIA2-1 | Dunnett's test | - | - | 0.0029 |
|  | sgCtrl vs sgGRIA2-2 | Dunnett's test | - | - | 0.006 |
| Fig 4H (MKN74) | Group | One-way ANOVA | F = 31.34 | (3, 8) | <0.0001 |
|  | ctrl vs sgGRIA2 | Tukey's HSD | - | - | 0.0008 |
|  | sgGRIA2 vs sgGRIA2+oeWT | Tukey's HSD | - | - | 0.0001 |
|  | sgGRIA2 vs sgGRIA2+oeR715A | Tukey's HSD | - | - | 0.3574 |
| Fig S4I (MKN45) | Group | One-way ANOVA | F = 36.1 | (3, 8) | <0.0001 |
|  | ctrl vs sgGRIA2 | Tukey's HSD | - | - | 0.0011 |
|  | sgGRIA2 vs sgGRIA2+oeWT | Tukey's HSD | - | - | <0.0001 |
|  | sgGRIA2 vs sgGRIA2+oeR715A | Tukey's HSD | - | - | 0.6264 |
| Fig 4I (MKN74) | Group | One-way ANOVA | F = 36.75 | (3, 8) | <0.0001 |
|  | ctrl vs sgGRIA2 | Tukey's HSD | - | - | 0.0237 |
|  | sgGRIA2 vs sgGRIA2+oeWT | Tukey's HSD | - | - | <0.0001 |
|  | sgGRIA2 vs sgGRIA2+oeR715A | Tukey's HSD | - | - | 0.4666 |
| Fig S4J (MKN45) | Group | One-way ANOVA | F = 69.6 | (3, 8) | <0.0001 |
|  | ctrl vs sgGRIA2 | Tukey's HSD | - | - | 0.0289 |
|  | sgGRIA2 vs sgGRIA2+oeWT | Tukey's HSD | - | - | <0.0001 |
|  | sgGRIA2 vs sgGRIA2+oeR715A | Tukey's HSD | - | - | 0.5366 |
| Fig 4K (MKN74) | Group | One-way ANOVA | F = 16.83 | (7, 16) | <0.0001 |
|  | ctrl vs sgGRIA2 | Tukey's HSD | - | - | 0.5504 |
|  | sgGRIA2 vs sgGRIA2+oeGRIA2WT | Tukey's HSD | - | - | 0.3031 |
|  | sgGRIA2 vs sgGRIA2+oeGRIA2Q607R | Tukey's HSD | - | - | 0.5516 |
|  | ctrl Ca2+ vs sgGRIA2 Ca2+ | Tukey's HSD | - | - | 0.0049 |
|  | sgGRIA2 Ca2+ vs sgGRIA2+oeGRIA2WT Ca2+ | Tukey's HSD | - | - | <0.0001 |
|  | sgGRIA2 Ca2+ vs sgGRIA2+oeGRIA2Q607R Ca2+ | Tukey's HSD | - | - | 0.3909 |
| Fig S4N (MKN45) | Group | One-way ANOVA | F = 23.37 | (7, 16) | <0.0001 |
|  | ctrl vs sgGRIA2 | Tukey's HSD | - | - | 0.9662 |
|  | sgGRIA2 vs sgGRIA2+oeGRIA2WT | Tukey's HSD | - | - | 0.3594 |
|  | sgGRIA2 vs sgGRIA2+oeGRIA2Q607R | Tukey's HSD | - | - | 0.1562 |
|  | ctrl Ca2+ vs sgGRIA2 Ca2+ | Tukey's HSD | - | - | 0.0004 |
|  | sgGRIA2 Ca2+ vs sgGRIA2+oeGRIA2WT Ca2+ | Tukey's HSD | - | - | <0.0001 |
|  | sgGRIA2 Ca2+ vs sgGRIA2+oeGRIA2Q607R Ca2+ | Tukey's HSD | - | - | 0.4153 |
| Fig 4L (MKN74) | Group | One-way ANOVA | F = 24.94 | (7, 16) | <0.0001 |
|  | ctrl vs sgGRIA2 | Tukey's HSD | - | - | 0.8300 |
|  | sgGRIA2 vs sgGRIA2+oeGRIA2WT | Tukey's HSD | - | - | 0.8276 |
|  | sgGRIA2 vs sgGRIA2+oeGRIA2Q607R | Tukey's HSD | - | - | 0.6714 |
|  | ctrl Ca2+ vs sgGRIA2 Ca2+ | Tukey's HSD | - | - | 0.0003 |
|  | sgGRIA2 Ca2+ vs sgGRIA2+oeGRIA2WT Ca2+ | Tukey's HSD | - | - | <0.0001 |
|  | sgGRIA2 Ca2+ vs sgGRIA2+oeGRIA2Q607R Ca2+ | Tukey's HSD | - | - | 0.5668 |
| Fig S4O (MKN45) | Group | One-way ANOVA | F = 38.79 | (7, 16) | <0.0001 |
|  | ctrl vs sgGRIA2 | Tukey's HSD | - | - | >0.9999 |
|  | sgGRIA2 vs sgGRIA2+oeGRIA2WT | Tukey's HSD | - | - | 0.5863 |
|  | sgGRIA2 vs sgGRIA2+oeGRIA2Q607R | Tukey's HSD | - | - | 0.9646 |
|  | ctrl Ca2+ vs sgGRIA2 Ca2+ | Tukey's HSD | - | - | 0.0011 |
|  | sgGRIA2 Ca2+ vs sgGRIA2+oeGRIA2WT Ca2+ | Tukey's HSD | - | - | <0.0001 |
|  | sgGRIA2 Ca2+ vs sgGRIA2+oeGRIA2Q607R Ca2+ | Tukey's HSD | - | - | 0.5283 |
| Fig 5A (MKN74) | Group | One-way ANOVA | F = 16.04 | (4, 10) | 0.0002 |
|  | ctrl vs NBQX 30uM | Dunnett's test | - | - | 0.0024 |
|  | ctrl vs NBQX 50uM | Dunnett's test | - | - | <0.0001 |
|  | ctrl vs Selurampanel 100nM | Dunnett's test |  |  | 0.0202 |
|  | ctrl vs Selurampanel 200nM | Dunnett's test |  |  | 0.0004 |
| Fig 5B (MKN74) | Group | One-way ANOVA | F = 21.47 | (4, 10) | <0.0001 |
|  | ctrl vs NBQX 30uM | Dunnett's test | - | - | 0.0004 |
|  | ctrl vs NBQX 50uM | Dunnett's test | - | - | <0.0001 |
|  | ctrl vs Selurampanel 100nM | Dunnett's test |  |  | 0.0208 |
|  | ctrl vs Selurampanel 200nM | Dunnett's test |  |  | 0.0001 |
| Fig 5C (MKN74) | Group | One-way ANOVA | F = 32.58 | (4, 10) | <0.0001 |
|  | ctrl vs NBQX 30uM | Dunnett's test | - | - | 0.0010 |
|  | ctrl vs NBQX 50uM | Dunnett's test | - | - | <0.0001 |
|  | ctrl vs Selurampanel 100nM | Dunnett's test |  |  | 0.0086 |
|  | ctrl vs Selurampanel 200nM | Dunnett's test |  |  | <0.0001 |
| Fig 5D (MKN74) | Group | One-way ANOVA | F = 16.34 | (4, 10) | 0.0002 |
|  | ctrl vs NBQX 30uM | Dunnett's test | - | - | 0.0019 |
|  | ctrl vs NBQX 50uM | Dunnett's test | - | - | <0.0001 |
|  | ctrl vs Selurampanel 100nM | Dunnett's test |  |  | 0.0088 |
|  | ctrl vs Selurampanel 200nM | Dunnett's test |  |  | 0.0003 |
| Fig S5A (MKN45) | Group | One-way ANOVA | F = 23.18 | (4, 10) | <0.0001 |
|  | ctrl vs NBQX 30uM | Dunnett's test | - | - | 0.0021 |
|  | ctrl vs NBQX 50uM | Dunnett's test | - | - | <0.0001 |
|  | ctrl vs Selurampanel 100nM | Dunnett's test |  |  | 0.0004 |
|  | ctrl vs Selurampanel 200nM | Dunnett's test |  |  | 0.0001 |
| Fig S5B (MKN45) | Group | One-way ANOVA | F = 12.61 | (4, 10) | 0.0006 |
|  | ctrl vs NBQX 30uM | Dunnett's test | - | - | 0.0243 |
|  | ctrl vs NBQX 50uM | Dunnett's test | - | - | 0.0002 |
|  | ctrl vs Selurampanel 100nM | Dunnett's test |  |  | 0.0072 |
|  | ctrl vs Selurampanel 200nM | Dunnett's test |  |  | 0.0012 |
| Fig S5C (MKN45) | Group | One-way ANOVA | F = 26.27 | (4, 10) | <0.0001 |
|  | ctrl vs NBQX 30uM | Dunnett's test | - | - | 0.0014 |
|  | ctrl vs NBQX 50uM | Dunnett's test | - | - | <0.0001 |
|  | ctrl vs Selurampanel 100nM | Dunnett's test |  |  | 0.0004 |
|  | ctrl vs Selurampanel 200nM | Dunnett's test |  |  | <0.0001 |
| Fig S5D (MKN45) | Group | One-way ANOVA | F = 26.33 | (4, 10) | <0.0001 |
|  | ctrl vs NBQX 30uM | Dunnett's test | - | - | 0.0010 |
|  | ctrl vs NBQX 50uM | Dunnett's test | - | - | <0.0001 |
|  | ctrl vs Selurampanel 100nM | Dunnett's test |  |  | 0.0045 |
|  | ctrl vs Selurampanel 200nM | Dunnett's test |  |  | <0.0001 |
| Fig 5I (MKN74, intensity) | Group | One-way ANOVA | F = 15.78 | (2, 15) | 0.0002 |
|  | ctrl vs NBQX | Dunnett's test | - | - | 0.0002 |
|  | ctrl vs Selurampanel | Dunnett's test | - | - | 0.0016 |
| Fig 5I (MKN45, intensity) | Group | One-way ANOVA | F = 64.66 | (2, 15) | <0.0001 |
|  | ctrl vs NBQX | Dunnett's test | - | - | <0.0001 |
|  | ctrl vs Selurampanel | Dunnett's test | - | - | <0.0001 |
| Fig 5I (MKN74, nodules) | Group | One-way ANOVA | F = 10.46 | (2, 15) | 0.0014 |
|  | ctrl vs NBQX | Dunnett's test | - | - | 0.0009 |
|  | ctrl vs Selurampanel | Dunnett's test | - | - | 0.011 |
| Fig 5I (MKN45, nodules | Group | One-way ANOVA | F = 61.51 | (2, 15) | <0.0001 |
|  | ctrl vs NBQX | Dunnett's test | - | - | <0.0001 |
|  | ctrl vs Selurampanel | Dunnett's test | - | - | <0.0001 |
| Fig 5J (GluR-2-HIGH, intensity) | Group | One-way ANOVA | F = 46.97 | (2, 15) | <0.0001 |
|  | ctrl vs NBQX | Dunnett's test | - | - | <0.0001 |
|  | ctrl vs Selurampanel | Dunnett's test | - | - | <0.0001 |
| Fig 5J (GluR-2-LOW, intensity) | Group | One-way ANOVA | F = 0.6878 | (2, 15) | 0.5178 |
|  | ctrl vs NBQX | Dunnett's test | - | - | 0.8639 |
|  | ctrl vs Selurampanel | Dunnett's test | - | - | 0.7048 |
| Fig 5J (GluR-2-HIGH, nodules) | Group | One-way ANOVA | F = 16.7 | (2, 15) | 0.0002 |
|  | ctrl vs NBQX | Dunnett's test | - | - | <0.0001 |
|  | ctrl vs Selurampanel | Dunnett's test | - | - | 0.0029 |
| Fig 5J (GluR-2-LOW, nodules) | Group | One-way ANOVA | F = 0.4188 | (2, 15) | 0.6653 |
|  | ctrl vs NBQX | Dunnett's test | - | - | 0.6512 |
|  | ctrl vs Selurampanel | Dunnett's test | - | - | >0.9999 |
| Fig 6D | Group | One-way ANOVA | F = 30.36 | (4, 10) | <0.0001 |
|  | Fibroblast vs Neutrophil | Dunnett's test | - | - | 0.0001 |
|  | Fibroblast vs Epithelial | Dunnett's test | - | - | <0.0001 |
|  | Fibroblast vs DC | Dunnett's test | - | - | <0.0001 |
|  | Fibroblast vs Endothelial | Dunnett's test | - | - | <0.0001 |
| Fig 6F | Overall (Interaction) | Two-way ANOVA | F = 14.67 | (1, 8) | 0.0050 |
|  | vec+WT vs oeGRIA2+WT | Bonferroni post hoc | - | - | 0.0019 |
|  | vec+CKO vs oeGRIA2+CKO | Bonferroni post hoc | - | - | >0.9999 |
|  | oeGRIA2+WT vs oeGRIA2+CKO | Bonferroni post hoc | - | - | 0.0004 |
| Fig 6G | Overall (Interaction) | Two-way ANOVA | F = 16.78 | (1, 8) | 0.0035 |
|  | vec+WT vs oeGRIA2+WT | Bonferroni post hoc | - | - | 0.0044 |
|  | vec+CKO vs oeGRIA2+CKO | Bonferroni post hoc | - | - | >0.9999 |
|  | oeGRIA2+WT vs oeGRIA2+CKO | Bonferroni post hoc | - | - | <0.0001 |
| Fig 6H | Overall (Interaction) | Two-way ANOVA | F = 35.07 | (1, 8) | 0.0004 |
|  | vec+WT vs oeGRIA2+WT | Bonferroni post hoc | - | - | 0.0003 |
|  | vec+CKO vs oeGRIA2+CKO | Bonferroni post hoc | - | - | >0.9999 |
|  | oeGRIA2+WT vs oeGRIA2+CKO | Bonferroni post hoc | - | - | <0.0001 |
| Fig 6I | Overall (Interaction) | Two-way ANOVA | F = 23.98 | (1, 8) | 0.0012 |
|  | vec+WT vs oeGRIA2+WT | Bonferroni post hoc | - | - | 0.0006 |
|  | vec+CKO vs oeGRIA2+CKO | Bonferroni post hoc | - | - | >0.9999 |
|  | oeGRIA2+WT vs oeGRIA2+CKO | Bonferroni post hoc | - | - | <0.0001 |
| Fig 6L (intensity) | Overall (Interaction) | Two-way ANOVA | F = 52.63 | (1, 20) | <0.0001 |
|  | vec+WT vs oeGRIA2+WT | Bonferroni post hoc | - | - | <0.0001 |
|  | vec+CKO vs oeGRIA2+CKO | Bonferroni post hoc | - | - | >0.9999 |
|  | oeGRIA2+WT vs oeGRIA2+CKO | Bonferroni post hoc | - | - | <0.0001 |
|  | vec+WT vs vec+CKO | Bonferroni post hoc | - | - | <0.0001 |
| Fig 6L (nodules) | Overall (Interaction) | Two-way ANOVA | F = 15.92 | (1, 20) | 0.0007 |
|  | vec+WT vs oeGRIA2+WT | Bonferroni post hoc | - | - | <0.0001 |
|  | vec+CKO vs oeGRIA2+CKO | Bonferroni post hoc | - | - | >0.9999 |
|  | oeGRIA2+WT vs oeGRIA2+CKO | Bonferroni post hoc | - | - | <0.0001 |
|  | vec+WT vs vec+CKO | Bonferroni post hoc | - | - | 0.0002 |
| Fig S6B | Group | One-way ANOVA | F = 84.94 | (4, 10) | <0.0001 |
|  | Fibroblast vs Neutrophil | Dunnett's test | - | - | <0.0001 |
|  | Fibroblast vs Epithelial | Dunnett's test | - | - | <0.0001 |
|  | Fibroblast vs DC | Dunnett's test | - | - | <0.0001 |
|  | Fibroblast vs Endothelial | Dunnett's test | - | - | <0.0001 |
| Fig S6D | WT vs CKO | Unpaired t-test | t=8.008 | 4 | 0.0013 |
| Fig 7D (GluR-2) | GluR-2 LOW vs HIGH | Mann-Whitney U test | U = 0 | - | <0.0001 |
| Fig 7D (β-cat) | GluR-2 LOW vs HIGH | Mann-Whitney U test | U = 10 | - | <0.0001 |
| Fig 7D (p-GSK3βS9) | GluR-2 LOW vs HIGH | Mann-Whitney U test | U = 22 | - | <0.0001 |

**Supplementary Table S5**

Antibodies used for fluorescence-activated cell sorting (FACS)

| **Marker** | **Conjugate** | **Catalog No.** | **Source** |
| --- | --- | --- | --- |
| CD45 | BV605 | 406-0459-42 | eBioscience |
| EpCAM | PE | 12-9326-42 | eBioscience |
| CD31 | APC | 17-0319-42 | eBioscience |
| PDGFRα | FITC | MA528585 | Invitrogen |
| HLA-DR | BV421 | 404-9956-42 | eBioscience |
| CD11b | PE-Cy7 | 25-0118-42 | eBioscience |
| CD15 | PE | 12-0159-42 | eBioscience |
| CD66b | APC | 17-0666-42 | eBioscience |
| CD3 | FITC | 11-0038-42 | eBioscience |
| CD14 | FITC | 11-0149-42 | eBioscience |
| CD19 | FITC | 11-0199-42 | eBioscience |
| CD56 | FITC | 11-0566-42 | eBioscience |
| Fixable Viability Dye | eFluor 780 | 65-0865-14 | eBioscience |

Note: CD3, CD14, CD19, and CD56 were combined as the FITC-conjugated lineage cocktail for dendritic cell identification.
